# Global variation in diabetes diagnosis and prevalence based on fasting glucose and hemoglobin A1c

**DOI:** 10.1038/s41591-023-02610-2

**Published:** 2023-11-09

**Authors:** NCD Risk Factor Collaboration (NCD-RisC), Bin Zhou, Kate E. Sheffer, James E. Bennett, Edward W. Gregg, Goodarz Danaei, Rosie K. Singleton, Jonathan E. Shaw, Anu Mishra, Victor P. F. Lhoste, Rodrigo M. Carrillo-Larco, Andre P. Kengne, Nowell H. Phelps, Rachel A. Heap, Archie W. Rayner, Gretchen A. Stevens, Chris J. Paciorek, Leanne M. Riley, Melanie J. Cowan, Stefan Savin, Stephen Vander Hoorn, Yuan Lu, Meda E. Pavkov, Giuseppina Imperatore, Carlos A. Aguilar-Salinas, Noor Ani Ahmad, Ranjit Mohan Anjana, Kairat Davletov, Farshad Farzadfar, Clicerio González-Villalpando, Young-Ho Khang, Hyeon Chang Kim, Tiina Laatikainen, Avula Laxmaiah, Jean Claude N. Mbanya, K. M. Venkat Narayan, Ambady Ramachandran, Alisha N. Wade, Tomasz Zdrojewski, Mohsen Abbasi-Kangevari, Hanan F. Abdul Rahim, Niveen M. Abu-Rmeileh, Shalkar Adambekov, Robert J. Adams, Wichai Aekplakorn, Imelda A. Agdeppa, Javad Aghazadeh-Attari, Charles Agyemang, Ali Ahmadi, Naser Ahmadi, Nastaran Ahmadi, Soheir H. Ahmed, Kamel Ajlouni, Halima Al-Hinai, Badreya Al-Lahou, Jawad A. Al-Lawati, Deena Al Asfoor, Nawal M. Al Qaoud, Monira Alarouj, Fadia AlBuhairan, Shahla AlDhukair, Maryam A. Aldwairji, Mohamed M. Ali, Farbod Alinezhad, Abdullah Alkandari, Husam F. Alomirah, Eman Aly, Deepak N. Amarapurkar, Lars Bo Andersen, Sigmund A. Anderssen, Dolores S. Andrade, Alireza Ansari-Moghaddam, Hajer Aounallah-Skhiri, Tahir Aris, Nimmathota Arlappa, Krishna K. Aryal, Felix K. Assah, Batyrbek Assembekov, Juha Auvinen, Mária Avdičová, Kishwar Azad, Mohsen Azimi-Nezhad, Fereidoun Azizi, Flora Bacopoulou, Nagalla Balakrishna, Mohamed Bamoshmoosh, Maciej Banach, Piotr Bandosz, José R. Banegas, Carlo M. Barbagallo, Alberto Barceló, Maja Baretić, Lena Barrera, Abdul Basit, Anwar M. Batieha, Aline P. Batista, Louise A. Baur, Antonisamy Belavendra, Habiba Ben Romdhane, Mikhail Benet, Salim Berkinbayev, Antonio Bernabe-Ortiz, Ximena Berrios Carrasola, Heloísa Bettiol, Augustin F. Beybey, Santosh K. Bhargava, Elysée Claude Bika Lele, Mukharram M. Bikbov, Bihungum Bista, Peter Bjerregaard, Espen Bjertness, Marius B. Bjertness, Cecilia Björkelund, Katia V. Bloch, Anneke Blokstra, Simona Bo, Martin Bobak, Jose G. Boggia, Marialaura Bonaccio, Alice Bonilla-Vargas, Herman Borghs, Pascal Bovet, Imperia Brajkovich, Hermann Brenner, Lizzy M. Brewster, Garry R. Brian, Yajaira Briceño, Miguel Brito, Anna Bugge, Frank Buntinx, Antonio Cabrera de León, Roberta B. Caixeta, Günay Can, Ana Paula C. Cândido, Mario V. Capanzana, Naděžda Čapková, Eduardo Capuano, Rocco Capuano, Vincenzo Capuano, Viviane C. Cardoso, Axel C. Carlsson, Felipe F. Casanueva, Laura Censi, Marvin Cervantes‐Loaiza, Parinya Chamnan, Snehalatha Chamukuttan, Queenie Chan, Fadi J. Charchar, Nish Chaturvedi, Huashuai Chen, Bahman Cheraghian, María-Dolores Chirlaque, Jerzy Chudek, Renata Cifkova, Massimo Cirillo, Frank Claessens, Emmanuel Cohen, Hans Concin, Cyrus Cooper, Simona Costanzo, Chris Cowell, Ana B. Crujeiras, Juan J. Cruz, Felipe V. Cureau, Sarah Cuschieri, Graziella D’Arrigo, Eleonora d’Orsi, Jean Dallongeville, Albertino Damasceno, Saeed Dastgiri, Amalia De Curtis, Giovanni de Gaetano, Stefaan De Henauw, Mohan Deepa, Vincent DeGennaro, Stefaan Demarest, Elaine Dennison, Valérie Deschamps, Meghnath Dhimal, Zivka Dika, Shirin Djalalinia, Chiara Donfrancesco, Guanghui Dong, Maria Dorobantu, Marcus Dörr, Nico Dragano, Wojciech Drygas, Yong Du, Charmaine A. Duante, Priscilla Duboz, Anar Dushpanova, Elzbieta Dziankowska-Zaborszczyk, Narges Ebrahimi, Ricky Eddie, Ebrahim Eftekhar, Vasiliki Efthymiou, Eruke E. Egbagbe, Sareh Eghtesad, Mohammad El-Khateeb, Jalila El Ati, Denise Eldemire-Shearer, Roberto Elosua, Ofem Enang, Rajiv T. Erasmus, Raimund Erbel, Cihangir Erem, Gul Ergor, Louise Eriksen, Johan G. Eriksson, Ali Esmaeili, Roger G. Evans, Ildar Fakhradiyev, Caroline H. Fall, Elnaz Faramarzi, Mojtaba Farjam, Yosef Farzi, Mohammad Reza Fattahi, Asher Fawwad, Francisco J. Felix-Redondo, Trevor S. Ferguson, Daniel Fernández-Bergés, Marika Ferrari, Catterina Ferreccio, Haroldo S. Ferreira, Eldridge Ferrer, Edith J. M. Feskens, David Flood, Maria Forsner, Sandrine Fosse, Edward F. Fottrell, Heba M. Fouad, Damian K. Francis, Guillermo Frontera, Takuro Furusawa, Zbigniew Gaciong, Sarah P. Garnett, Magda Gasull, Andrea Gazzinelli, Ulrike Gehring, Ebrahim Ghaderi, Seyyed-Hadi Ghamari, Ali Ghanbari, Erfan Ghasemi, Oana-Florentina Gheorghe-Fronea, Anup Ghimire, Alessandro Gialluisi, Simona Giampaoli, Francesco Gianfagna, Tiffany K. Gill, Glen Gironella, Aleksander Giwercman, David Goltzman, Aleksandra Gomula, Helen Gonçalves, Mauer Gonçalves, David A. Gonzalez-Chica, Marcela Gonzalez-Gross, Juan P. González-Rivas, María-Elena González-Villalpando, Angel R. Gonzalez, Frederic Gottrand, Dušan Grafnetter, Tomasz Grodzicki, Anders Grøntved, Ramiro Guerrero, Unjali P. Gujral, Rajeev Gupta, Laura Gutierrez, Xinyi Gwee, Rosa Haghshenas, Hamid Hakimi, Ian R. Hambleton, Behrooz Hamzeh, Willem A. Hanekom, Dominique Hange, Sari Hantunen, Jie Hao, Rachakulla Hari Kumar, Javad Harooni, Seyed Mohammad Hashemi-Shahri, Jun Hata, Christin Heidemann, Rafael dos Santos Henrique, Sauli Herrala, Karl-Heinz Herzig, Ramin Heshmat, Sai Yin Ho, Michelle Holdsworth, Reza Homayounfar, Wilma M. Hopman, Andrea R. V. R. Horimoto, Claudia Hormiga, Bernardo L. Horta, Leila Houti, Christina Howitt, Thein Thein Htay, Aung Soe Htet, Maung Maung Than Htike, José María Huerta, Ilpo Tapani Huhtaniemi, Martijn Huisman, Abdullatif Husseini, Inge Huybrechts, Licia Iacoviello, Ellina M. Iakupova, Anna G. Iannone, Norazizah Ibrahim Wong, Chinwuba Ijoma, Vilma E. Irazola, Takafumi Ishida, Godsent C. Isiguzo, Sheikh Mohammed Shariful Islam, Duygu Islek, Till Ittermann, Masanori Iwasaki, Tuija Jääskeläinen, Jeremy M. Jacobs, Hashem Y. Jaddou, Michel Jadoul, Bakary Jallow, Kenneth James, Kazi M. Jamil, Edward Janus, Marjo-Riitta Jarvelin, Grazyna Jasienska, Ana Jelaković, Bojan Jelaković, Garry Jennings, Anjani Kumar Jha, Ramon O. Jimenez, Karl-Heinz Jöckel, Jari J. Jokelainen, Jost B. Jonas, Pradeep Joshi, Josipa Josipović, Farahnaz Joukar, Jacek Jóźwiak, Anthony Kafatos, Eero O. Kajantie, Zhanna Kalmatayeva, Khem B. Karki, Marzieh Katibeh, Jussi Kauhanen, Gyulli M. Kazakbaeva, François F. Kaze, Calvin Ke, Sirkka Keinänen-Kiukaanniemi, Roya Kelishadi, Maryam Keramati, Mathilde Kersting, Yousef Saleh Khader, Arsalan Khaledifar, Davood Khalili, Bahareh Kheiri, Motahareh Kheradmand, Alireza Khosravi, Ursula Kiechl-Kohlendorfer, Sophia J. Kiechl, Stefan Kiechl, Andrew Kingston, Heidi Klakk, Jana Klanova, Michael Knoflach, Patrick Kolsteren, Jürgen König, Raija Korpelainen, Paul Korrovits, Jelena Kos, Seppo Koskinen, Sudhir Kowlessur, Slawomir Koziel, Susi Kriemler, Peter Lund Kristensen, Daan Kromhout, Ruzena Kubinova, Urho M. Kujala, Mukhtar Kulimbet, Pawel Kurjata, Catherine Kyobutungi, Quang Ngoc La, Demetre Labadarios, Carl Lachat, Youcef Laid, Lachmie Lall, Tiina Lankila, Vera Lanska, Georg Lappas, Bagher Larijani, Tint Swe Latt, Martino Laurenzi, Nils Lehmann, Terho Lehtimäki, Daniel Lemogoum, Gabriel M. Leung, Yanping Li, M. Fernanda Lima-Costa, Hsien-Ho Lin, Lars Lind, Lauren Lissner, Xiaotian Liu, Esther Lopez-Garcia, Tania Lopez, José Eugenio Lozano, Dalia Luksiene, Annamari Lundqvist, Nuno Lunet, Michala Lustigová, George L. L. Machado-Coelho, Aristides M. Machado-Rodrigues, Enguerran Macia, Luisa M. Macieira, Ahmed A. Madar, Gladys E. Maestre, Stefania Maggi, Dianna J. Magliano, Emmanuella Magriplis, Gowri Mahasampath, Bernard Maire, Marcia Makdisse, Mohammad-Reza Malekpour, Fatemeh Malekzadeh, Reza Malekzadeh, Kodavanti Mallikharjuna Rao, Sofia Malyutina, Lynell V. Maniego, Yannis Manios, Masimango Imani Mannix, Fariborz Mansour-Ghanaei, Enzo Manzato, Paula Margozzini, Joany Mariño, Larissa Pruner Marques, Reynaldo Martorell, Luis P. Mascarenhas, Masoud Masinaei, Ellisiv B. Mathiesen, Tandi E. Matsha, Anselmo J. Mc Donald Posso, Shelly R. McFarlane, Stephen T. McGarvey, Sounnia Mediene Benchekor, Kirsten Mehlig, Amir Houshang Mehrparvar, Jesus D. Melgarejo, Fabián Méndez, Ana Maria B. Menezes, Alibek Mereke, Indrapal I. Meshram, Diane T. Meto, Cláudia S. Minderico, G. K. Mini, Juan Francisco Miquel, J. Jaime Miranda, Mohammad Reza Mirjalili, Pietro A. Modesti, Sahar Saeedi Moghaddam, Mostafa K. Mohamed, Kazem Mohammad, Mohammad Reza Mohammadi, Zahra Mohammadi, Noushin Mohammadifard, Reza Mohammadpourhodki, Viswanathan Mohan, Muhammad Fadhli Mohd Yusoff, Iraj Mohebbi, Niels C. Møller, Dénes Molnár, Amirabbas Momenan, Charles K. Mondo, Roger A. Montenegro Mendoza, Eric Monterrubio-Flores, Mahmood Moosazadeh, Farhad Moradpour, Alain Morejon, Luis A. Moreno, Karen Morgan, Suzanne N. Morin, Alireza Moslem, Mildrey Mosquera, Malgorzata Mossakowska, Aya Mostafa, Seyed-Ali Mostafavi, Mohammad Esmaeel Motlagh, Jorge Motta, Kelias P. Msyamboza, Thet Thet Mu, Maria L. Muiesan, Jaakko Mursu, Kamarul Imran Musa, Norlaila Mustafa, Muel Telo M. C. Muyer, Iraj Nabipour, Gabriele Nagel, Balkish M. Naidu, Farid Najafi, Jana Námešná, Vinay B. Nangia, Take Naseri, Nareemarn Neelapaichit, Azim Nejatizadeh, Ilona Nenko, Flavio Nervi, Tze Pin Ng, Chung T. Nguyen, Quang Ngoc Nguyen, Michael Y. Ni, Peng Nie, Ramfis E. Nieto-Martínez, Toshiharu Ninomiya, Marianna Noale, Oscar A. Noboa, Davide Noto, Mohannad Al Nsour, Irfan Nuhoğlu, Terence W. O’Neill, Augustine N. Odili, Kyungwon Oh, Ryutaro Ohtsuka, Mohd Azahadi Omar, Altan Onat, Sok King Ong, Obinna Onodugo, Pedro Ordunez, Rui Ornelas, Pedro J. Ortiz, Clive Osmond, Afshin Ostovar, Johanna A. Otero, Charlotte B. Ottendahl, Akaninyene Otu, Ellis Owusu-Dabo, Luigi Palmieri, Wen-Harn Pan, Songhomitra Panda-Jonas, Francesco Panza, Mariela Paoli, Suyeon Park, Mahboubeh Parsaeian, Nikhil D. Patel, Raimund Pechlaner, Ivan Pećin, João M. Pedro, Sergio Viana Peixoto, Markku Peltonen, Alexandre C. Pereira, Thaliane Mayara Pessôa dos Prazeres, Niloofar Peykari, Modou Cheyassin Phall, Son Thai Pham, Hiep Hoang Phan, Rafael N. Pichardo, Hynek Pikhart, Aida Pilav, Pavel Piler, Freda Pitakaka, Aleksandra Piwonska, Andreia N. Pizarro, Pedro Plans-Rubió, Silvia Plata, Miquel Porta, Anil Poudyal, Farhad Pourfarzi, Akram Pourshams, Hossein Poustchi, Rajendra Pradeepa, Rui Providencia, Jardena J. Puder, Solie Puhakka, Margus Punab, Mostafa Qorbani, Hedley K. Quintana, Tran Quoc Bao, Salar Rahimikazerooni, Olli Raitakari, Manuel Ramirez-Zea, Jacqueline Ramke, Rafel Ramos, Lekhraj Rampal, Sanjay Rampal, Daniel A. Rangel Reina, Mohammad-Mahdi Rashidi, Josep Redon, Jane D. P. Renner, Cézane P. Reuter, Luis Revilla, Negar Rezaei, Abbas Rezaianzadeh, Fernando Rigo, Reina G. Roa, Louise Robinson, Fernando Rodríguez-Artalejo, María del Cristo Rodriguez-Perez, Laura A. Rodríguez-Villamizar, Andrea Y. Rodríguez, Ulla Roggenbuck, Peter Rohloff, Elisabetta L. Romeo, Annika Rosengren, Adolfo Rubinstein, Petra Rust, Marcin Rutkowski, Hamideh Sabbaghi, Harshpal S. Sachdev, Alireza Sadjadi, Ali Reza Safarpour, Sare Safi, Saeid Safiri, Mohammad Hossien Saghi, Olfa Saidi, Nader Saki, Sanja Šalaj, Benoit Salanave, Jukka T. Salonen, Massimo Salvetti, Jose Sánchez-Abanto, Diana A. Santos, Lèlita C. Santos, Maria Paula Santos, Tamara R. Santos, Jouko L. Saramies, Luis B. Sardinha, Nizal Sarrafzadegan, Kai-Uwe Saum, Mariana Sbaraini, Marcia Scazufca, Beatriz D. Schaan, Christa Scheidt-Nave, Sabine Schipf, Carsten O. Schmidt, Ben Schöttker, Sara Schramm, Sylvain Sebert, Moslem Sedaghattalab, Aye Aye Sein, Sadaf G. Sepanlou, Ronel Sewpaul, Teresa Shamah-Levy, Seyed Morteza Shamshirgaran, Maryam Sharafkhah, Sanjib K. Sharma, Almaz Sharman, Amaneh Shayanrad, Ali Akbar Shayesteh, Hana Shimizu-Furusawa, Rahman Shiri, Namuna Shrestha, Khairil Si-Ramlee, Diego Augusto Santos Silva, Mary Simon, Judith Simons, Leon A. Simons, Michael Sjöström, Jolanta Slowikowska-Hilczer, Przemysław Slusarczyk, Liam Smeeth, Eugène Sobngwi, Stefan Söderberg, Agustinus Soemantri, Reecha Sofat, Vincenzo Solfrizzi, Mohammad Hossein Somi, Aïcha Soumaré, Alfonso Sousa-Poza, Karen Sparrenberger, Jan A. Staessen, Bill Stavreski, Jostein Steene-Johannessen, Peter Stehle, Aryeh D. Stein, Jochanan Stessman, Jakub Stokwiszewski, Karien Stronks, Milton F. Suarez-Ortegón, Phalakorn Suebsamran, Johan Sundström, Paibul Suriyawongpaisal, René Charles Sylva, Moyses Szklo, Abdonas Tamosiunas, Mohammed Rasoul Tarawneh, Carolina B. Tarqui-Mamani, Anne Taylor, Julie Taylor, Tania Tello, K. R. Thankappan, Holger Theobald, Xenophon Theodoridis, Nihal Thomas, Amanda G. Thrift, Erik J. Timmermans, Dwi Hapsari Tjandrarini, Hanna K. Tolonen, Janne S. Tolstrup, Maciej Tomaszewski, Murat Topbas, Laura Torres-Collado, Pierre Traissac, Areti Triantafyllou, John Tuitele, Azaliia M. Tuliakova, Marshall K. Tulloch-Reid, Tomi-Pekka Tuomainen, Evangelia Tzala, Christophe Tzourio, Peter Ueda, Eunice Ugel, Flora A. M. Ukoli, Hanno Ulmer, Hannu M. T. Uusitalo, Gonzalo Valdivia, Bert-Jan van den Born, Johan Van der Heyden, Hoang Van Minh, Lenie van Rossem, Natasja M. Van Schoor, Irene G. M. van Valkengoed, Elisabeth M. van Zutphen, Dirk Vanderschueren, Diego Vanuzzo, Senthil K. Vasan, Tomas Vega, Gustavo Velasquez-Melendez, Roosmarijn Verstraeten, Lucie Viet, Salvador Villalpando, Jesus Vioque, Jyrki K. Virtanen, Bharathi Viswanathan, Ari Voutilainen, Wan Mohamad Wan Bebakar, Wan Nazaimoon Wan Mohamud, Chongjian Wang, Ningli Wang, Qian Wang, Ya Xing Wang, Ying-Wei Wang, S. Goya Wannamethee, Karen Webster-Kerr, Niels Wedderkopp, Wenbin Wei, Leo D. Westbury, Peter H. Whincup, Kurt Widhalm, Indah S. Widyahening, Andrzej Więcek, Rainford J. Wilks, Johann Willeit, Peter Willeit, Tom Wilsgaard, Bogdan Wojtyniak, Andrew Wong, Emily B. Wong, Mark Woodward, Frederick C. Wu, Haiquan Xu, Liang Xu, Nor Azwany Yaacob, Li Yan, Weili Yan, Moein Yoosefi, Akihiro Yoshihara, Novie O. Younger-Coleman, Yu-Ling Yu, Yunjiang Yu, Ahmad Faudzi Yusoff, Ahmad A. Zainuddin, Farhad Zamani, Sabina Zambon, Antonis Zampelas, Ko Ko Zaw, Tajana Zeljkovic Vrkic, Yi Zeng, Zhen-Yu Zhang, Bekbolat Zholdin, Paul Zimmet, Emanuel Zitt, Nada Zoghlami, Julio Zuñiga Cisneros, Majid Ezzati

**Affiliations:** 1 Imperial College London, London, UK; 2 RCSI University of Medicine and Health Sciences, Dublin, Ireland; 3 Harvard T. H. Chan School of Public Health, Boston, MA USA; 4 Baker Heart and Diabetes Institute, Melbourne, Victoria Australia; 5 Bill & Melinda Gates Foundation, Seattle, WA USA; 6 Emory University, Atlanta, GA USA; 7 South African Medical Research Council, Cape Town, South Africa; 8 World Health Organization, Geneva, Switzerland; 9 University of California Berkeley, Berkeley, CA USA; 10 University of Auckland, Auckland, New Zealand; 11 Yale School of Public Health, New Haven, CT USA; 12 US Centres for Disease Control and Prevention, Atlanta, GA USA; 13 Instituto Nacional de Ciencias Medicas y Nutricion, Mexico City, Mexico; 14 Ministry of Health, Kuala Lumpur, Malaysia; 15 Madras Diabetes Research Foundation, Chennai, India; 16 Asfendiyarov Kazakh National Medical University, Almaty, Kazakhstan; 17 Tehran University of Medical Sciences, Tehran, Iran; 18 National Institute of Public Health, Cuernavaca, Mexico; 19 Seoul National University College of Medicine, Seoul, Republic of Korea; 20 Yonsei University College of Medicine, Seoul, Republic of Korea; 21 Finnish Institute for Health and Welfare, Helsinki, Finland; 22 ICMR - National Institute of Nutrition, Hyderabad, India; 23 University of Yaoundé 1, Yaoundé, Cameroon; 24 India Diabetes Research Foundation, Chennai, India; 25 University of the Witwatersrand, Johannesburg, South Africa; 26 Medical University of Gdansk, Gdansk, Poland; 27 Non-Communicable Diseases Research Center, Tehran, Iran; 28 Qatar University, Doha, Qatar; 29 Birzeit University, Birzeit, State of Palestine; 30 Al-Farabi Kazakh National University, Almaty, Kazakhstan; 31 Flinders University, Adelaide, South Australia Australia; 32 Mahidol University, Nakhon Pathom, Thailand; 33 Food and Nutrition Research Institute, Taguig, The Philippines; 34 Urmia University of Medical Sciences, Urmia, Iran; 35 University of Amsterdam, Amsterdam, The Netherlands; 36 Modeling in Health Research Center, Shahrekord, Iran; 37 Shahid Sadoughi University of Medical Sciences, Yazd, Iran; 38 University of Oslo, Oslo, Norway; 39 The National Center for Diabetes, Endocrinology and Genetics, Amman, Jordan; 40 Ministry of Health, Muscat, Oman; 41 Kuwait Institute for Scientific Research, Kuwait City, Kuwait; 42 World Health Organization Regional Office for the Eastern Mediterranean, Cairo, Egypt; 43 Ministry of Health, Kuwait City, Kuwait; 44 Dasman Diabetes Institute, Kuwait City, Kuwait; 45 Aldara Hospital and Medical Center, Riyadh, Saudi Arabia; 46 King Abdullah International Medical Research Center, Riyadh, Saudi Arabia; 47 Tabriz University of Medical Sciences, Tabriz, Iran; 48 Bombay Hospital and Medical Research Centre, Mumbai, India; 49 Western Norway University of Applied Sciences, Sogndal, Norway; 50 Norwegian School of Sport Sciences, Oslo, Norway; 51 Universidad de Cuenca, Cuenca, Ecuador; 52 Zahedan University of Medical Sciences, Zahedan, Iran; 53 National Institute of Public Health, Tunis, Tunisia; 54 University of Bergen, Bergen, Norway; 55 Oulu University Hospital, Oulu, Finland; 56 University of Oulu, Oulu, Finland; 57 Regional Authority of Public Health, Banska Bystrica, Slovakia; 58 Diabetic Association of Bangladesh, Dhaka, Bangladesh; 59 Neyshabur University of Medical Sciences, Neyshabur, Iran; 60 Research Institute for Endocrine Sciences, Tehran, Iran; 61 National and Kapodistrian University of Athens, Athens, Greece; 62 University of Science and Technology, Sana’a, Yemen; 63 Medical University of Lodz, Lodz, Poland; 64 Universidad Autónoma de Madrid CIBERESP, Madrid, Spain; 65 University of Palermo, Palermo, Italy; 66 University of Miami, Miami, FL USA; 67 University Hospital Centre Zagreb, Zagreb, Croatia; 68 Universidad del Valle, Cali, Colombia; 69 Baqai Institute of Diabetology and Endocrinology, Karachi, Pakistan; 70 Jordan University of Science and Technology, Irbid, Jordan; 71 Universidade Federal de Ouro Preto, Ouro Preto, Brazil; 72 University of Sydney, Sydney, New South Wales Australia; 73 Christian Medical College Vellore, Vellore, India; 74 University Tunis El Manar, Tunis, Tunisia; 75 Cafam University Foundation, Bogotá, Colombia; 76 Kazakh National Medical University, Almaty, Kazakhstan; 77 Universidad Peruana Cayetano Heredia, Lima, Peru; 78 Pontificia Universidad Católica de Chile, Santiago, Chile; 79 University of São Paulo, São Paulo, Brazil; 80 Sunder Lal Jain Hospital, Delhi, India; 81 Institute of Medical Research and Medicinal Plant Studies, Yaoundé, Cameroon; 82 Ufa Eye Research Institute, Ufa, Russia; 83 Nepal Health Research Council, Kathmandu, Nepal; 84 University of Southern Denmark, Copenhagen, Denmark; 85 University of Gothenburg, Gothenburg, Sweden; 86 Universidade Federal do Rio de Janeiro, Rio de Janeiro, Brazil; 87 National Institute for Public Health and the Environment, Bilthoven, The Netherlands; 88 University of Turin, Turin, Italy; 89 University College London, London, UK; 90 Universidad de la República, Montevideo, Uruguay; 91 IRCCS Neuromed, Pozzilli, Italy; 92 Caja Costarricense de Seguro Social, San José, Costa Rica; 93 KU Leuven, Leuven, Belgium; 94 Ministry of Health, Victoria, Seychelles; 95 Unisanté, Lausanne, Switzerland; 96 Universidad Central de Venezuela, Caracas, Venezuela; 97 German Cancer Research Center, Heidelberg, Germany; 98 The Fred Hollows Foundation, Auckland, New Zealand; 99 University of the Andes, Mérida, Venezuela; 100 Instituto Politécnico de Lisboa, Lisbon, Portugal; 101 University College Copenhagen, Copenhagen, Denmark; 102 Universidad de La Laguna, Tenerife, Spain; 103 Pan American Health Organization, Washington, DC USA; 104 Istanbul University - Cerrahpasa, Istanbul, Türkiye; 105 Universidade Federal de Juiz de Fora, Juiz de Fora, Brazil; 106 National Institute of Public Health, Prague, Czech Republic; 107 Gaetano Fucito Hospital, Mercato San Severino, Italy; 108 Karolinska Institutet, Huddinge, Sweden; 109 Santiago de Compostela University, Santiago de Compostela, Spain; 110 Council for Agricultural Research and Economics, Rome, Italy; 111 Sanpasitthiprasong Regional Hospital, Ubon Ratchathani, Thailand; 112 Federation University Australia, Ballarat, Victoria Australia; 113 Xiangtan University, Xiangtan, China; 114 Ahvaz Jundishapur University of Medical Sciences, Ahvaz, Iran; 115 CIBERESP, Madrid, Spain; 116 Medical University of Silesia, Katowice, Poland; 117 Charles University, Prague, Czech Republic; 118 Thomayer University Hospital, Prague, Czech Republic; 119 University of Salerno, Fisciano, Italy; 120 UMR CNRS-MNHN 7206, Paris, France; 121 Agency for Preventive and Social Medicine, Bregenz, Austria; 122 University of Southampton, Southampton, UK; 123 CIBEROBN, Madrid, Spain; 124 Universidade Federal do Rio Grande do Norte, Natal, Brazil; 125 University of Malta, Msida, Malta; 126 National Research Council, Reggio Calabria, Italy; 127 Federal University of Santa Catarina, Florianópolis, Brazil; 128 Institut Pasteur de Lille, Lille, France; 129 Eduardo Mondlane University, Maputo, Mozambique; 130 Tabriz Health Services Management Research Center, Tabriz, Iran; 131 Ghent University, Ghent, Belgium; 132 Innovating Health International, Port-au-Prince, Haiti; 133 Sciensano, Brussels, Belgium; 134 French Public Health Agency, St Maurice, France; 135 University of Zagreb, Zagreb, Croatia; 136 Ministry of Health and Medical Education, Tehran, Iran; 137 Istituto Superiore di Sanità, Rome, Italy; 138 Sun Yat-sen University, Guangzhou, China; 139 Carol Davila University of Medicine and Pharmacy, Bucharest, Romania; 140 University Medicine Greifswald, Greifswald, Germany; 141 University Hospital Düsseldorf, Düsseldorf, Germany; 142 Lazarski University, Warsaw, Poland; 143 Robert Koch Institute, Berlin, Germany; 144 IRL 3189 ESS, Marseille, France; 145 Scuola Superiore Sant’Anna, Pisa, Italy; 146 Ministry of Health and Medical Services, Gizo, Solomon Islands; 147 Hormozgan University of Medical Sciences, Bandar Abbas, Iran; 148 University of Benin, Benin City, Nigeria; 149 National Institute of Nutrition and Food Technology, Tunis, Tunisia; 150 The University of the West Indies, Kingston, Jamaica; 151 Institut Hospital del Mar d’Investigacions Mèdiques, Barcelona, Spain; 152 CIBERCV, Barcelona, Spain; 153 University of Calabar, Calabar, Nigeria; 154 University of Stellenbosch, Cape Town, South Africa; 155 University of Duisburg-Essen, Essen, Germany; 156 Karadeniz Technical University, Trabzon, Türkiye; 157 Dokuz Eylul University, Izmir, Türkiye; 158 University of Helsinki, Helsinki, Finland; 159 Rafsanjan University of Medical Sciences, Rafsanjan, Iran; 160 Monash University, Melbourne, Victoria Australia; 161 Fasa University of Medical Sciences, Fasa, Iran; 162 Shiraz University of Medical Sciences, Shiraz, Iran; 163 Baqai Medical University, Karachi, Pakistan; 164 Centro de Salud Villanueva Norte, Badajoz, Spain; 165 Hospital Don Benito-Villanueva de la Serena, Badajoz, Spain; 166 Federal University of Alagoas, Maceió, Brazil; 167 Wageningen University, Wageningen, The Netherlands; 168 Wuqu’ Kawoq, Tecpan, Guatemala; 169 Umeå University, Umeå, Sweden; 170 Hospital Universitario Son Espases, Palma, Spain; 171 Kyoto University, Kyoto, Japan; 172 Medical University of Warsaw, Warsaw, Poland; 173 Universidade Federal de Minas Gerais, Belo Horizonte, Brazil; 174 Utrecht University, Utrecht, The Netherlands; 175 Kurdistan University of Medical Sciences, Sanandaj, Iran; 176 B. P. Koirala Institute of Health Sciences, Dharan, Nepal; 177 University of Insubria, Varese, Italy; 178 Mediterranea Cardiocentro, Naples, Italy; 179 University of Adelaide, Adelaide, South Australia Australia; 180 Lund University, Lund, Sweden; 181 McGill University, Montreal, Québec Canada; 182 PASs Hirszfeld Institute of Immunology and Experimental Therapy, Wroclaw, Poland; 183 Federal University of Pelotas, Pelotas, Brazil; 184 University Agostinho Neto, Luanda, Angola; 185 Universidad Politécnica de Madrid, Madrid, Spain; 186 International Clinical Research Center, Brno, Czech Republic; 187 Centro de Estudios en Diabetes A.C, Mexico City, Mexico; 188 Universidad Autónoma de Santo Domingo, Santo Domingo, Dominican Republic; 189 University of Lille, Lille, France; 190 Institute for Clinical and Experimental Medicine, Prague, Czech Republic; 191 Jagiellonian University Medical College, Kraków, Poland; 192 University of Southern Denmark, Odense, Denmark; 193 Universidad Icesi, Cali, Colombia; 194 Eternal Heart Care Centre and Research Institute, Jaipur, India; 195 Institute for Clinical Effectiveness and Health Policy, Buenos Aires, Argentina; 196 National University of Singapore, Singapore, Singapore; 197 The University of the West Indies, Cave Hill, Barbados; 198 Kermanshah University of Medical Sciences, Kermanshah, Iran; 199 Africa Health Research Institute, Durban, South Africa; 200 University of Eastern Finland, Kuopio, Finland; 201 Capital Medical University, Beijing, China; 202 Yasuj University of Medical Sciences, Yasuj, Iran; 203 Kyushu University, Fukuoka, Japan; 204 Federal University of Pernambuco, Recife, Brazil; 205 Chronic Diseases Research Center, Tehran, Iran; 206 University of Hong Kong, Hong Kong, China; 207 French National Research Institute for Sustainable Development, Montpellier, France; 208 Shahid Beheshti University of Medical Sciences, Tehran, Iran; 209 Kingston Health Sciences Centre, Kingston, Ontario Canada; 210 Universidad Autónoma de Bucaramanga, Bucaramanga, Colombia; 211 University Oran 1, Oran, Algeria; 212 Independent Public Health Specialist, Nay Pyi Taw, Myanmar; 213 Ministry of Health and Sports, Nay Pyi Taw, Myanmar; 214 VU University Medical Center, Amsterdam, The Netherlands; 215 International Agency for Research on Cancer, Lyon, France; 216 College of Medicine, University of Nigeria, Ituku-Ozalla, Enugu, Nigeria; 217 The University of Tokyo, Tokyo, Japan; 218 Alex Ekwueme Federal University Teaching Hospital, Abakaliki, Nigeria; 219 Deakin University, Geelong, Victoria Australia; 220 Hokkaido University, Sapporo, Japan; 221 Hadassah University Medical Center, Jerusalem, Israel; 222 Université Catholique de Louvain, Brussels, Belgium; 223 Gambia National Nutrition Agency, Banjul, The Gambia; 224 Kuwait Institute for Scientific Research, Safat, Kuwait; 225 University of Melbourne, Melbourne, Victoria Australia; 226 Heart Foundation, Melbourne, Victoria Australia; 227 Universidad Eugenio Maria de Hostos, Santo Domingo, Dominican Republic; 228 Institute of Molecular and Clinical Ophthalmology Basel, Basel, Switzerland; 229 World Health Organization Country Office, Delhi, India; 230 Guilan University of Medical Sciences, Rasht, Iran; 231 University of Opole, Opole, Poland; 232 University of Crete, Heraklion, Greece; 233 Maharajgunj Medical Campus, Kathmandu, Nepal; 234 Aarhus University, Aarhus, Denmark; 235 University of Toronto, Toronto, Ontario, Canada; 236 Research Institute for Primordial Prevention of Non-communicable Disease, Isfahan, Iran; 237 Mashhad University of Medical Sciences, Mashhad, Iran; 238 Research Institute of Child Nutrition, Dortmund, Germany; 239 Shahrekord University of Medical Sciences, Shahrekord, Iran; 240 Mazandaran University of Medical Sciences, Sari, Iran; 241 Hypertension Research Center, Isfahan, Iran; 242 Medical University of Innsbruck, Innsbruck, Austria; 243 VASCage - Research Centre on Vascular Ageing and Stroke, Innsbruck, Austria; 244 Newcastle University, Newcastle, UK; 245 University College South Denmark, Haderslev, Denmark; 246 Masaryk University, Brno, Czech Republic; 247 University of Vienna, Vienna, Austria; 248 Tartu University Clinics, Tartu, Estonia; 249 Ministry of Health and Wellness, Port Louis, Mauritius; 250 University of Zurich, Zurich, Switzerland; 251 University of Groningen, Groningen, The Netherlands; 252 University of Jyväskylä, Jyväskylä, Finland; 253 National Institute of Cardiology, Warsaw, Poland; 254 African Population and Health Research Center, Nairobi, Kenya; 255 Hanoi University of Public Health, Hanoi, Vietnam; 256 University of Limpopo, Polokwane, South Africa; 257 Stellennbosch University, Polokwane, South Africa; 258 Ministry of Health, Algiers, Algeria; 259 Ministry of Health, Georgetown, Guyana; 260 Oulu Deaconess Institute Foundation, Oulu, Finland; 261 Sahlgrenska Academy, Gothenburg, Sweden; 262 Endocrinology and Metabolism Research Center, Tehran, Iran; 263 University of Public Health, Yangon, Myanmar; 264 Centro Studi Epidemiologici di Gubbio, Gubbio, Italy; 265 Tampere University Hospital, Tampere, Finland; 266 Tampere University, Tampere, Finland; 267 University of Douala, Douala, Cameroon; 268 Oswaldo Cruz Foundation Rene Rachou Research Institute, Belo Horizonte, Brazil; 269 National Taiwan University, Taipei, Taiwan; 270 Uppsala University, Uppsala, Sweden; 271 Zhengzhou University, Zhengzhou, China; 272 Universidad San Martín de Porres, Lima, Peru; 273 Consejería de Sanidad Junta de Castilla y León, Valladolid, Spain; 274 Lithuanian University of Health Sciences, Kaunas, Lithuania; 275 University of Porto, Porto, Portugal; 276 University of Coimbra, Coimbra, Portugal; 277 Coimbra University Hospital Center, Coimbra, Portugal; 278 University of Texas Rio Grande Valley, Harlingen, TX USA; 279 Institute of Neuroscience of the National Research Council, Padua, Italy; 280 Agricultural University of Athens, Athens, Greece; 281 Academia VBHC, São Paulo, Brazil; 282 Institute of Internal and Preventive Medicine, Novosibirsk, Russia; 283 Harokopio University, Athens, Greece; 284 Université Catholique de Bukavu, Bukavu, Democratic Republic of the Congo; 285 University of Padua, Padua, Italy; 286 Secretaria de Estado da Saúde de Santa Catarina, Florianópolis, Brazil; 287 Universidade Estadual do Centro-Oeste, Guarapuava, Brazil; 288 UiT The Arctic University of Norway, Tromsø, Norway; 289 Sefako Makgatho Health Sciences University, Pretoria, South Africa; 290 Instituto Conmemorativo Gorgas de Estudios de la Salud, Panama City, Panama; 291 Brown University, Providence, RI USA; 292 University of Abidjan, Abidjan, Côte d’Ivoire; 293 Universidade de Lisboa, Lisbon, Portugal; 294 Saveetha Institute of Medical and Technical Sciences, Chennai, India; 295 Università degli Studi di Firenze, Florence, Italy; 296 Ain Shams University, Cairo, Egypt; 297 Psychiatry and Psychology Research Center, Tehran, Iran; 298 Isfahan Cardiovascular Research Center, Isfahan, Iran; 299 University of Pécs, Pécs, Hungary; 300 Mulago Hospital, Kampala, Uganda; 301 Gorgas Memorial Institute for Studies of Health, Panama City, Panama; 302 University of Medical Sciences of Cienfuegos, Cienfuegos, Cuba; 303 University of Zaragoza, Zaragoza, Spain; 304 Sabzevar University of Medical Sciences, Sabzevar, Iran; 305 International Institute of Molecular and Cell Biology, Warsaw, Poland; 306 World Health Organization Country Office, Lilongwe, Malawi; 307 Department of Public Health, Nay Pyi Taw, Myanmar; 308 University of Brescia, Brescia, Italy; 309 Universiti Sains Malaysia, Kelantan, Malaysia; 310 Universiti Kebangsaan Malaysia, Kuala Lumpur, Malaysia; 311 University de Kinshasa, Kinshasa, Democratic Republic of the Congo; 312 Bushehr University of Medical Sciences, Bushehr, Iran; 313 Ulm University, Ulm, Germany; 314 Department of Statistics, Kuala Lumpur, Malaysia; 315 Suraj Eye Institute, Nagpur, India; 316 Ministry of Health, Apia, Samoa; 317 Mahidol University, Bangkok, Thailand; 318 National Institute of Hygiene and Epidemiology, Hanoi, Vietnam; 319 Hanoi Medical University, Hanoi, Vietnam; 320 Xi’an Jiaotong University, Xi’an, China; 321 Precision Care Clinic Corp, St. Cloud, FL USA; 322 Eastern Mediterranean Public Health Network, Amman, Jordan; 323 University of Manchester, Manchester, UK; 324 University of Abuja College of Health Sciences, Abuja, Nigeria; 325 Korea Disease Control and Prevention Agency, Cheongju-si, Republic of Korea; 326 Japan Wildlife Research Center, Tokyo, Japan; 327 Istanbul University, Istanbul, Türkiye; 328 Ministry of Health, Bandar Seri Begawan, Brunei; 329 University of Madeira, Funchal, Portugal; 330 Osteoporosis Research Center, Tehran, Iran; 331 Universidad de Santander, Bucaramanga, Colombia; 332 Kwame Nkrumah University of Science and Technology, Kumasi, Ghana; 333 Academia Sinica, Taipei, Taiwan; 334 Privatpraxis Prof Jonas und Dr Panda-Jonas, Heidelberg, Germany; 335 IRCCS Ente Ospedaliero Specializzato in Gastroenterologia S. de Bellis, Bari, Italy; 336 Jivandeep Hospital, Anand, India; 337 Centro de Investigação em Saúde de Angola, Caxito, Angola; 338 Vietnam National Heart Institute, Hanoi, Vietnam; 339 National Hospital of Endocrinology, Hanoi, Vietnam; 340 Clínica de Medicina Avanzada Dr. Abel González, Santo Domingo, Dominican Republic; 341 University of Sarajevo, Sarajevo, Bosnia and Herzegovina; 342 Ministry of Health and Medical Services, Honiara, Solomon Islands; 343 Public Health Agency of Catalonia, Barcelona, Spain; 344 Observatorio de Salud Pública de Santander, Bucaramanga, Colombia; 345 Ardabil University of Medical Sciences, Ardabil, Iran; 346 Lausanne University Hospital, Lausanne, Switzerland; 347 Alborz University of Medical Sciences, Karaj, Iran; 348 Ministry of Health, Hanoi, Vietnam; 349 University of Turku, Turku, Finland; 350 Institute of Nutrition of Central America and Panama, Guatemala City, Guatemala; 351 Institut Universitari d’Investigació en Atenció Primària Jordi Gol, Girona, Spain; 352 Universiti Putra Malaysia, Serdang, Malaysia; 353 University of Malaya, Kuala Lumpur, Malaysia; 354 University of Valencia, Valencia, Spain; 355 University of Santa Cruz do Sul, Santa Cruz do Sul, Brazil; 356 CS S. Agustín Ibsalut, Palma, Spain; 357 Ministerio de Salud, Panama City, Panama; 358 Canarian Health Service, Tenerife, Spain; 359 Universidad Industrial de Santander, Bucaramanga, Colombia; 360 Ministery of Health and Social Protection, Bogotá, Colombia; 361 Associazione Calabrese di Epatologia, Reggio Calabria, Italy; 362 Sahlgrenska University Hospital, Gothenburg, Sweden; 363 Sitaram Bhartia Institute of Science and Research, New Delhi, India; 364 University or Zagreb, Zagreb, Croatia; 365 National Institute of Health, Lima, Peru; 366 Wellbeing Services County of South Karelia, Lappeenranta, Finland; 367 Universidade Federal do Rio Grande do Sul, Porto Alegre, Brazil; 368 University of São Paulo Clinics Hospital, São Paulo, Brazil; 369 Human Sciences Research Council, Cape Town, South Africa; 370 Academy of Preventive Medicine, Almaty, Kazakhstan; 371 Teikyo University, Tokyo, Japan; 372 Finnish Institute of Occupational Health, Helsinki, Finland; 373 Public Health Promotion and Development Organization, Kathmandu, Nepal; 374 St Vincent’s Hospital, Sydney, New South Wales Australia; 375 University of New South Wales, Sydney, New South Wales Australia; 376 Karolinska Institutet, Stockholm, Sweden; 377 London School of Hygiene & Tropical Medicine, London, UK; 378 Diponegoro University, Semarang, Indonesia; 379 University of Bari, Bari, Italy; 380 University of Bordeaux, Bordeaux, France; 381 University of Hohenheim, Stuttgart, Germany; 382 Bonn University, Bonn, Germany; 383 National Institute of Public Health - National Institute of Hygiene, Warsaw, Poland; 384 Pontificia Universidad Javeriana Seccional Cali, Cali, Colombia; 385 Ubon Ratchathani University, Ubon Ratchathani, Thailand; 386 National Statistical Office, Praia, Cabo Verde; 387 Johns Hopkins Bloomberg School of Public Health, Baltimore, MD USA; 388 Ministry of Health, Amman, Jordan; 389 Amrita Institute of Medical Sciences, Kochi, India; 390 Aristotle University of Thessaloniki, Thessaloniki, Greece; 391 University Medical Center Utrecht, Utrecht, The Netherlands; 392 National Research and Innovation Agency, Jakarta, Indonesia; 393 Universidad Miguel Hernandez, Madrid, Spain; 394 Department of Health, Faga’alu, American Samoa; 395 LBJ Hospital, Faga’alu, American Samoa; 396 Universidad Centro-Occidental Lisandro Alvarado, Barquisimeto, Venezuela; 397 Meharry Medical College, Nashville, TN USA; 398 University of Tampere Tays Eye Center, Tampere, Finland; 399 MONICA-FRIULI Study Group, Udine, Italy; 400 Institute of Tropical Medicine, Antwerp, Belgium; 401 CIBERESP, Alicante, Spain; 402 Institute for Medical Research, Kuala Lumpur, Malaysia; 403 Capital Medical University Beijing Tongren Hospital, Beijing, China; 404 Xinjiang Medical University, Urumqi, China; 405 Ministry of Health and Welfare, Taipei, Taiwan; 406 The Ministry of Health and Wellness, Kingston, Jamaica; 407 St George’s, University of London, London, UK; 408 Medical University of Vienna, Vienna, Austria; 409 Universitas Indonesia, Jakarta, Indonesia; 410 Institute of Food and Nutrition Development of Ministry of Agriculture and Rural Affairs, Beijing, China; 411 Beijing Institute of Ophthalmology, Beijing, China; 412 Children’s Hospital of Fudan University, Shanghai, China; 413 Niigata University, Niigata, Japan; 414 South China Institute of Environmental Sciences, Guangzhou, China; 415 Iran University of Medical Sciences, Tehran, Iran; 416 Peking University, Beijing, China; 417 Duke University, Durham, NC USA; 418 West Kazakhstan Medical University, Aktobe, Kazakhstan; 419 University of Ghana, Accra, Ghana

**Keywords:** Diagnostic markers, Diabetes, Diagnosis, Public health, Epidemiology

## Abstract

Fasting plasma glucose (FPG) and hemoglobin A1c (HbA1c) are both used to diagnose diabetes, but these measurements can identify different people as having diabetes. We used data from 117 population-based studies and quantified, in different world regions, the prevalence of diagnosed diabetes, and whether those who were previously undiagnosed and detected as having diabetes in survey screening, had elevated FPG, HbA1c or both. We developed prediction equations for estimating the probability that a person without previously diagnosed diabetes, and at a specific level of FPG, had elevated HbA1c, and vice versa. The age-standardized proportion of diabetes that was previously undiagnosed and detected in survey screening ranged from 30% in the high-income western region to 66% in south Asia. Among those with screen-detected diabetes with either test, the age-standardized proportion who had elevated levels of both FPG and HbA1c was 29–39% across regions; the remainder had discordant elevation of FPG or HbA1c. In most low- and middle-income regions, isolated elevated HbA1c was more common than isolated elevated FPG. In these regions, the use of FPG alone may delay diabetes diagnosis and underestimate diabetes prevalence. Our prediction equations help allocate finite resources for measuring HbA1c to reduce the global shortfall in diabetes diagnosis and surveillance.

## Abstract (web summary)

Analysis of HbA1c and FPG levels across 117 population-based studies demonstrates regional variation in prevalence of previously undiagnosed screen-detected diabetes using one or both measures and suggests that use of elevated FPG alone could underestimate diabetes prevalence in low- and middle-income countries.

## Main

Diabetes is associated with debilitating complications such as amputation, vision loss and renal failure, and with increased risk of cardiovascular events, dementia, some cancers and infectious diseases such as severe COVID-19 and tuberculosis^1–6^. The diagnostic criteria for diabetes have evolved over time to incorporate hemoglobin A1c (HbA1c), which is a measure of long-term glycemic status and more convenient to measure for patients than fasting glucose or the 2-h oral glucose tolerance test (OGTT)^7–10^. In contemporary guidelines, any one or the combination of fasting plasma glucose (FPG), OGTT and HbA1c may be used to diagnose diabetes^10–14^. With the exception of diagnosis of gestational diabetes, OGTT is now rarely used in clinical practice or population surveillance because of the inconvenience related to the glucose load, 2-h time frame and the two blood draws required for the test^15,16^. FPG and HbA1c, which are both used in clinical practice and epidemiological research and surveillance, measure different glycemic features, namely basal glucose level (FPG) and average glucose level in the previous 2–3 months (HbA1c)^17^. Therefore, individuals may have elevated levels of one or both biomarkers, and FPG and HbA1c may classify different people as having diabetes^9,10^. Diabetes also has a long subclinical period defined by hyperglycemia and can remain undiagnosed without screening or other mechanisms for early identification^18^.

Some studies have assessed sensitivity and specificity of diabetes diagnosis using either FPG or HbA1c relative to the OGTT or have compared diabetes prevalence based on these different glycemic biomarkers, but most did not provide a direct comparison of HbA1c and FPG^19–21^. Most population-based studies on the concordance and discordance of diabetes diagnosis using FPG versus HbA1c have been conducted in a single country or region^14,22–42^ and the only multi-country study^43^ used data largely from high-income western countries. Therefore, there are scant data on how the concordance and discordance of FPG and HbA1c in classifying diabetes vary across regions in the world, and on the factors associated with this variation. The lack of data on the regional variation in diabetes identified based on FPG versus HbA1c means that we cannot quantify the full extent of the global diabetes epidemic and its regional variation, because diabetes prevalence is measured and reported using a single glycemic biomarker in most population-based surveys and analyses^44–46^. For example, in the latest global analysis^44^, only ~15% of surveys had measured both FPG and HbA1c.

We assembled a global database of population-based studies that had measured both FPG and HbA1c. Using these data, we quantified the regional variation in the extent of diabetes diagnosis, with diabetes defined as in the Methods. We also quantified, among those who were previously undiagnosed and were detected as having diabetes through screening in the survey, the concordance and discordance of having FPG and HbA1c above common diagnostic thresholds (7.0 mmol l^−1^ for FPG and 6.5% for HbA1c). We refer to this group as screen-detected diabetes, which is an epidemiological definition, because many clinical guidelines recommend two measurements for diabetes diagnosis^10–13^. We then used regression analysis to examine what individual and study-level factors were associated with whether participants with screen-detected diabetes were identified by elevated FPG, elevated HbA1c or elevated levels of both. It has been shown that having elevated levels of both biomarkers has high positive predictive value for subsequent clinical diagnosis and risk of complications^14,47^, and hence this group is similar to clinically diagnosed diabetes.

Finally, we leveraged the global coverage of the dataset and its large sample size to develop prediction equations that estimate, for any given FPG level, the probability that a person without previously diagnosed diabetes would have HbA1c above the clinical threshold for diabetes had it been measured, and vice versa. We aimed to develop and validate global and generalizable prediction equations that account for both personal characteristics and regional differences. These equations serve three purposes. First, they allow more efficient use of finite diagnostic resources, by identifying some people with below- or near-threshold level for one biomarker (for example, FPG) for measurement of another (for example, HbA1c). Second, they allow the estimation of the probability that a person with a screen-detected elevated level of one biomarker would also have an elevated level of the other, as a confirmation of diabetes status^14,47^. Finally, the prediction equations could improve diabetes surveillance by allowing estimation of prevalence of diabetes based on both FPG and HbA1c in health surveys that have measured only one of these biomarkers.

## Results

### Data sources

**Fig. 1:**
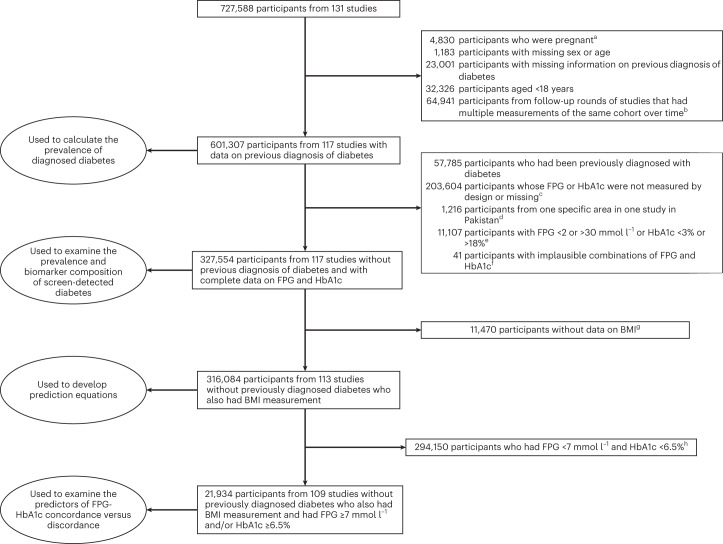
Flowchart of data cleaning and use. ^a^Excluded because glucose metabolism changes during pregnancy. ^b^Data from the first available measurement were used for these participants. ^c^Some surveys only measured glycemic biomarker on a subset of participants for logistic or budget reasons. ^d^Excluded because glycemic measurements in these participants were systematically different from the rest from the same study, possibly because the specific area had high prevalence of thalassemia^94^. ^e^Excluded because such values are more likely to be due to data recording error than values within the range. ^f^We removed participants for implausible pairs of FPG and HbA1c using the method of local outlier factor (LOF)^95^. This approach detects data combinations that are extremes in the joint density of the variable pairs (for example, a participant with FPG of 5 mmol l^−1^ and HbA1c of 17%, or with FPG of 28 mmol l^−1^ and HbA1c of 5%). We identified extremes as those measurements whose measure of local density by LOF method is less than half of the average of their 100 nearest neighbors. ^g^Including all 2,436 participants from four studies that did not measure BMI. ^h^Including all 3,455 participants from four studies in which all individuals without previously diagnosed diabetes had FPG < 7.0 mmol l^−1^ and HbA1c < 6.5%.

We used data collated by the NCD Risk Factor Collaboration (NCD-RisC), a global consortium of population-based health examination surveys and studies with measurement of both FPG and HbA1c, and with data on previous diagnosis of diabetes, as described in the Methods. The criteria for including and excluding studies are stated in Methods. Within each study, we excluded participants who had missing data or were pregnant, under 18 years of age or from follow-up rounds of studies that had multiple measurements of the same cohort over time (Fig. 1). After exclusions, we used data on 601,307 participants aged 18 years and older with information on whether they had been previously diagnosed with diabetes, of whom 364,825 participants also had measured FPG and HbA1c. The difference between the number of participants with data on previous diagnosis and with biomarker data is mostly because many studies do blood tests on a subsample of those with questionnaire data. These participants were from 117 studies whose mid-year was from 2000 to 2021 in 45 countries from seven of eight world regions (Extended Data Table 1). We had no study that measured both FPG and HbA1c from the region of Oceania, which consists of Pacific island nations. The number of studies in other regions ranged from seven in sub-Saharan Africa to 48 in the high-income western region (Table 1). The mean age of study participants was 50 years and 56% of participants were women. Of the 117 studies with data on glycemic variables, 113 (97%) with 351,270 participants (96% of all participants) also had data on body-mass index (BMI); the remaining four studies either did not collect anthropometric information or only had self-reported height and weight data.

**Table 1:** Characteristics of studies and participants included in the analysis: all participants, participants without diagnosed diabetes, and participants without diagnosed diabetes who had FPG ≥7.0 mmol l^−1^ and/or HbA1c ≥6.5%

	Number of studies	Number of countries (% of all countries in the region or world)	Median year of studies	Number of participants	Percent female (%)	Mean (s.d.) age (years)	Mean FPG (mmol l^−1^)	Mean HbA1c (%)	Mean BMI (kg m^−2^)
**All participants**									
Central and eastern Europe	8	4 (20%)	2012	51,352	55.6	55 (11)	5.8	5.5	28.2
Central Asia, Middle East and north Africa	10	5 (18%)	2015	73,109	54.4	47 (15)	5.7	5.9	27.7
High-income western	48	11 (41%)	2010	190,276	53.2	53 (18)	5.6	5.5	27.8
Latin America and the Caribbean	17	11 (31%)	2016	75,257	62.3	48 (18)	5.7	5.7	28.3
South Asia	8	2 (29%)	2012	87,404	54.4	42 (14)	5.9	6.0	23.1
East and southeast Asia and the Pacific	19	7 (41%)	2012	112,854	56.2	52 (16)	5.6	5.7	24.0
Sub-Saharan Africa	7	5 (10%)	2014	11,055	62.6	49 (14)	6.1	6.2	26.3
All studies	117	45 (22%)	2012	601,307	55.6	50 (17)	5.7	5.7	26.4
**Participants without diagnosed diabetes**									
Central and eastern Europe	8	4 (20%)	2012	12,086	52.2	49 (14)	5.4	5.4	27.4
Central Asia, Middle East and north Africa	10	5 (18%)	2015	46,886	55.1	46 (14)	5.3	5.6	27.5
High-income western	48	11 (41%)	2010	100,140	53.9	52 (16)	5.4	5.3	27.4
Latin America and the Caribbean	17	11 (31%)	2016	38,524	60.8	48 (17)	5.3	5.4	28.0
South Asia	8	2 (29%)	2012	28,554	52.7	41 (14)	5.6	5.7	24.0
East and southeast Asia and the Pacific	19	7 (41%)	2012	92,900	56.6	51 (16)	5.4	5.6	23.9
Sub-Saharan Africa	7	5 (10%)	2014	8,464	62.2	48 (14)	5.6	5.8	26.2
All studies	117	45 (22%)	2012	327,554	55.7	49 (16)	5.4	5.5	26.2
**Participants without diagnosed diabetes who had FPG** **≥** **7.0** **mmol** **l**^**−1**^ **and/or HbA1c** **≥** **6.5%**									
Central and eastern Europe	8	4 (20%)	2012	551	41.7	58 (11)	8.0	6.4	31.3
Central Asia, Middle East and north Africa	10	5 (18%)	2015	3,328	52.0	55 (13)	7.7	7.3	30.2
High-income western	44	11 (41%)	2009	4,422	43.1	62 (13)	7.9	6.7	31.0
Latin America and the Caribbean	17	11 (31%)	2016	2,718	63.0	55 (15)	8.4	7.3	30.4
South Asia	8	2 (29%)	2012	4,612	51.7	47 (13)	8.0	7.4	26.0
East and southeast Asia and the Pacific	19	7 (41%)	2012	6,157	52.0	58 (13)	8.1	7.0	26.1
Sub-Saharan Africa	7	5 (10%)	2014	1,257	60.5	55 (11)	7.5	7.2	28.7
All studies	113	45 (22%)	2013	23,045	51.7	56 (14)	8.0	7.1	28.4

### Screen-detected diabetes by FPG and HbA1c

**Fig. 2:**
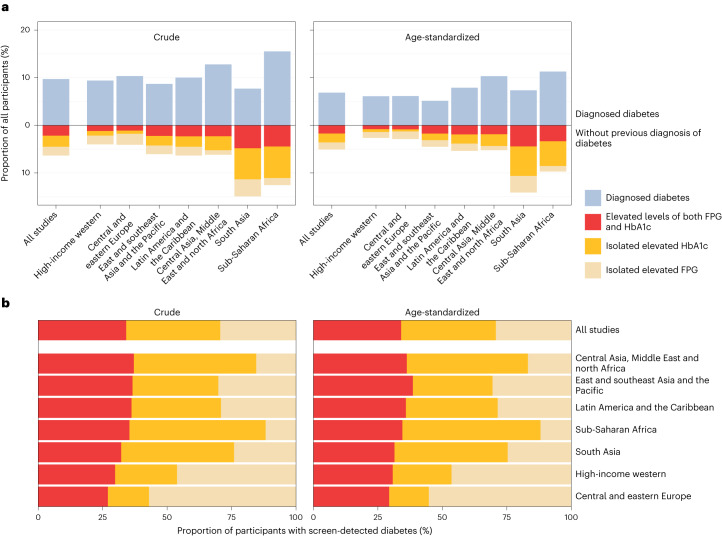
Extent and composition of diagnosed and screen-detected diabetes by region. **a**, Crude and age-standardized proportion of participants with diagnosed or screen-detected diabetes and, for those without previous diagnosis, whether they had isolated elevated FPG (FPG ≥ 7.0 mmol l^−1^ and HbA1c < 6.5%), isolated elevated HbA1c (HbA1c ≥ 6.5% and FPG < 7.0 mmol l^−1^) or elevated levels of both. **b**, Crude and age-standardized proportion of participants with screen-detected diabetes who had isolated elevated FPG, isolated elevated HbA1c or elevated levels of both, by region. The contents in **b** are the same as the segment of **a** that is below the zero line, scaled to 100% so that the composition of screen-detected diabetes can be compared across regions, regardless of its total prevalence. Having elevated levels of both biomarkers has high positive predictive value for subsequent clinical diagnosis and risk of complications^14,47^ and hence this group is similar to clinically diagnosed diabetes. In **a**, regions are ordered by the total proportion of participants who had diagnosed and screen-detected diabetes. In **b**, regions are ordered by the crude proportion of participants with screen-detected diabetes who had elevated levels of both FPG and HbA1c. Extended Data Fig. 1 provides sex-specific results.

Across all studies, 16% of participants had diagnosed or previously undiagnosed screen-detected diabetes. Diagnosed diabetes was calculated based on reporting a previous diagnosis and screen-detected diabetes as having FPG and/or HbA1c levels at or above the thresholds of 7.0 mmol l^−1^ and 6.5% (refs. ^10–13^) (Fig. 2). After age-standardization, the total prevalence of diabetes became 12%. The age-standardized prevalence of diagnosed and screen-detected diabetes were 7% and 5%, respectively. Those without a previous diabetes diagnosis had a lower BMI than those with a previous diagnosis in every region, by an average of 2.9 kg m^−2^ across all studies (Table 1). Among those without a previous diagnosis, participants with screen-detected diabetes (FPG ≥7.0 mmol l^−1^ and/or HbA1c ≥ 6.5%) had a mean BMI that was higher than those who did not have diabetes (FPG < 7.0 mmol l^−1^ and HbA1c < 6.5%) by an average of 2.4 kg m^−2^.

**Extended Data Fig. 2:**
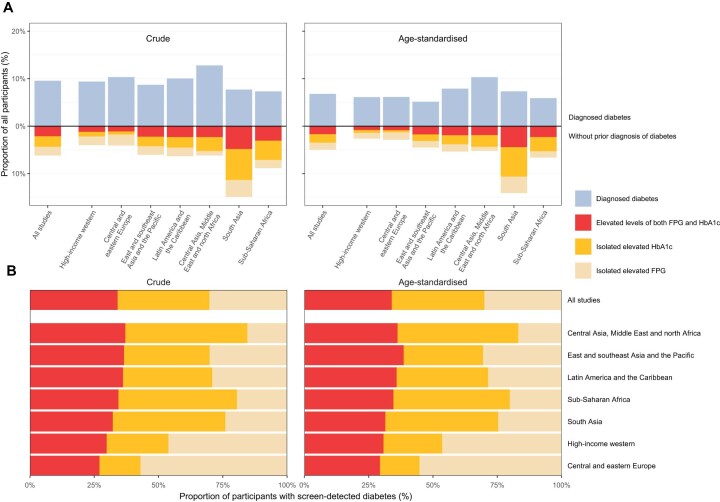
Extent and composition of diagnosed and screen-detected diabetes by region, after removing two studies in Mauritius from sub-Saharan Africa. (**a**) Crude and age-standardized proportion of participants with diagnosed or screen-detected diabetes, and, for those without prior diagnosis, whether they had isolated elevated FPG (FPG ≥7.0 mmol/L and HbA1c < 6.5%), isolated elevated HbA1c (HbA1c ≥6.5% and FPG < 7.0 mmol/L) or elevated levels of both, and (**b**) crude and age-standardized proportion of participants with screen-detected diabetes who had isolated elevated FPG, isolated elevated HbA1c or elevated levels of both, by region. Its contents are the same as the segment of Panel A that is below the zero line, scaled to 100% so that the composition of screen-detected diabetes can be compared across regions, regardless of its total prevalence. Having elevated levels of both biomarkers has high positive predictive value for subsequent clinical diagnosis and risk of complications^14,47^, and hence this group is similar to clinically-diagnosed diabetes. In panel A, regions are ordered by the total proportion of participants who had diagnosed and screen-detected diabetes. In panel B, regions are ordered by the crude proportion of participants with screen-detected diabetes who had elevated levels of both FPG and HbA1c. Regions are in the same order as in Fig. 2.

In most regions, age-standardized diabetes prevalence was slightly lower than crude prevalence, except south Asia where the participants were on average younger than in other regions (Table 1). Regionally, the age-standardized total diabetes prevalence (the combination of diagnosed and screen-detected diabetes) ranged from ~9% in the high-income western region to ~21% in south Asia and sub-Saharan Africa. The age-standardized proportion of diabetes that was previously undiagnosed, and was detected in the screening via the survey, was highest (66%) in studies from south Asia, and lowest (<35%) in studies from the high-income western region, central and eastern Europe, and the region of central Asia, Middle East and north Africa. Two studies in sub-Saharan Africa were from Mauritius, a country that is different demographically and economically from most other countries in the region. When these studies were removed, total age-standardized diabetes prevalence in sub-Saharan Africa declined from 21% to 13% and the proportion who were previously undiagnosed increased from 46% to 53% (Extended Data Fig. 2).

**Extended Data Fig. 3:**
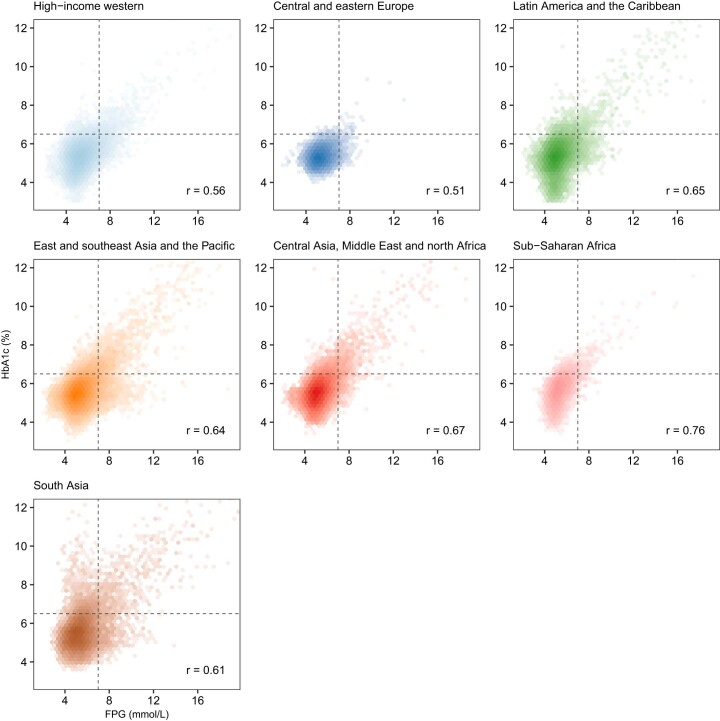
Relationship between FPG and HbA1c, among participants who had not been previously diagnosed with diabetes, by region. The shading indicates the density of participants in each region, with darker shades corresponding to more participants and vice versa. The dotted lines are placed at FPG of 7.0 mmol/L and HbA1c of 6.5%, which are common clinical thresholds for diabetes^10–13^. The numbers on the panels indicate the Pearson correlation coefficient between FPG and HbA1c in each region. A total of 623 (0.2%) participants with FPG of 19-28 mmol/L and/or HbA1c of 12-17% are not shown in the figure so that the axes have sufficient resolution in ranges where the great majority of participants were.

Across all studies together, 29% of participants with screen-detected diabetes had isolated elevated FPG, 37% had isolated elevated HbA1c and 34% had elevated levels of both. These global proportions were the same before and after age-standardization. There was substantial variation across regions in the composition of screen-detected diabetes across these three groups, both in terms of whether both biomarkers were elevated or only one, and in the case of the latter, whether the elevated biomarker was FPG or HbA1c (Fig. 2). Regionally, the shares of participants in these three groups changed little after age-standardization, and we report the age-standardized results here. The age-standardized proportion of those with screen-detected diabetes who had elevated levels of both FPG and HbA1c ranged from 29–39% across regions. The remaining 61–71% of participants with screen-detected diabetes had discordant FPG and HbA1c elevations. Isolated elevated HbA1c made up 54% of participants with screen-detected diabetes in sub-Saharan Africa, and 47% in the region of central Asia, Middle East and north Africa. In these regions, isolated elevated FPG accounted for <17% of all screen-detected diabetes. In contrast, 55% of participants with screen-detected diabetes in central and eastern Europe, and 46% in high-income western region, had isolated elevated FPG. The correlation coefficient between FPG and HbA1c among participants without previous diagnosis of diabetes ranged from 0.51 in central and eastern Europe to 0.76 in sub-Saharan Africa (Extended Data Fig. 3).

### Association with individual and study characteristics

Some participant and study-level characteristics were associated with whether screen-detected diabetes was manifested as elevated levels of FPG, HbA1c or both (Table 2). Among those with screen-detected diabetes, male sex was associated with a higher probability of having elevated FPG, either alone (prevalence ratio (PR) = 1.10; 95% credible interval (CrI) 1.07–1.14) or together with elevated HbA1c (1.07; 1.03–1.11), and with a lower probability of having isolated elevated HbA1c (0.86; 0.83–0.89). Older age was associated with a lower probability of having elevated FPG, alone (PR = 0.97 per decade of age; 0.96–0.98) or together with elevated HbA1c (PR = 0.97; 0.96–0.99) and a higher probability of having isolated elevated HbA1c (1.05; 1.04–1.06). Higher BMI was associated with a higher probability of having concordant elevation of FPG and HbA1c (PR = 1.07 per 5 units; 1.06–1.08) and a lower probability of having isolated elevated FPG (PR = 0.92; 0.90–0.93).

**Table 2:** Association of whether screen-detected diabetes is manifested as isolated elevated FPG, isolated elevated HbA1c or elevated levels of both with individual and study characteristics

	Isolated elevated FPG			Isolated elevated HbA1c			Elevated levels of both		
	PR	CrI	Posterior probability	PR	CrI	Posterior probability	PR	CrI	Posterior probability
Region									
High-income western	Reference			Reference			Reference		
Central and eastern Europe	1.16	0.73–1.86	0.259	0.62	0.35–1.09	0.049	0.83	0.61–1.12	0.115
Latin America and the Caribbean	0.48	0.32–0.72	<0.001	1.42	0.93–2.16	0.053	1.16	0.91–1.46	0.109
East and southeast Asia and the Pacific	0.51	0.35–0.73	<0.001	1.53	1.04–2.25	0.015	1.35	1.10–1.67	0.002
South Asia	0.24	0.13–0.44	<0.001	1.65	0.89–3.10	0.056	1.52	1.08–2.15	0.009
Central Asia, Middle East and north Africa	0.33	0.20–0.54	<0.001	2.20	1.31–3.67	0.001	1.06	0.80–1.40	0.342
Sub-Saharan Africa	0.33	0.19–0.57	<0.001	1.65	0.92–2.94	0.045	1.31	0.96–1.79	0.045
Sex									
Women	Reference			Reference			Reference		
Men	1.10	1.07–1.14	<0.001	0.86	0.83–0.89	<0.001	1.07	1.03-1.11	<0.001
Age (per 10 years of age)	0.97	0.96–0.98	<0.001	1.05	1.04–1.06	<0.001	0.97	0.96–0.99	<0.001
BMI (per 5 kg m^−2^)	0.92	0.90–0.93	<0.001	0.99	0.98–1.01	0.137	1.07	1.06–1.08	<0.001
Study year (per 5 years of time)	1.01	0.89–1.14	0.447	1.05	0.92–1.20	0.240	1.06	0.99–1.14	0.048
Percent people with diabetes who had been diagnosed before (per 10 percentage points)	0.98	0.89–1.09	0.380	0.98	0.88–1.09	0.354	1.05	0.99–1.11	0.046
Measurement of FPG									
Laboratory	Reference			Reference			Reference		
Portable device	1.71	1.00–2.91	0.025	0.89	0.51–1.56	0.338	0.87	0.64–1.16	0.169
Measurement of HbA1c									
Laboratory	Reference			Reference			Reference		
Portable device	0.33	0.16–0.68	0.001	2.13	1.05–4.20	0.018	0.54	0.35–0.81	0.002

The association with each variable is reported as prevalence ratios (PRs), adjusted for all other variables in the table, in the regression models described in the Methods, in which data from individual participants with screen-detected diabetes were used. Extended Data Table 7 shows results excluding studies that had measured FPG in capillary whole blood using a portable device. CrI, credible interval.

At the study level, in studies that used a portable device to measure HbA1c, the composition of screen-detected diabetes was shifted toward more isolated elevated HbA1c, but the estimates for this association had wide confidence intervals because the great majority of studies in our analysis had measured glucose and HbA1c in a laboratory. Neither the year of study nor the percentage of participants with diabetes who had reported previous diagnosis were associated with the composition of screen-detected diabetes.

After adjustment for participant and study characteristics, regional differences remained in the composition of screen-detected diabetes (Table 2). After adjustment for these factors, the composition of screen-detected diabetes, in terms of having elevated FPG and HbA1c in isolation or together, was statistically indistinguishable between the high-income western region and central and eastern Europe. In other regions, elevated HbA1c was a more common form of screen-detected diabetes than in the high-income western region, in isolation (PR ranging 1.42–2.20 across these regions) or together with elevated FPG (PR ranging 1.31–1.52 in east and southeast Asia and the Pacific; south Asia; sub-Saharan Africa). In all regions, isolated elevated FPG was less common than in the high-income western region (PR ranging 0.24–0.51).

### Prediction equations

We developed nine prediction equations (Extended Data Table 2) that estimate, for any given FPG level, the probability that a person without previously diagnosed diabetes would have HbA1c above the clinical threshold for diabetes had it been measured, and vice versa. The variables in the prediction equations included FPG as well as sex, age, BMI, whether FPG was measured in a laboratory or using a portable device, and region. We assessed the performance of the models in predicting (1) individual participants’ status of having HbA1c ≥ 6.5% based on their FPG and (2) the prevalence of HbA1c ≥ 6.5% for an entire study. We used the same method for predicting the probability of having FPG ≥ 7.0 mmol l^−1^ based on HbA1c. The performance at the individual level reflects how well the prediction equation works for triaging patients for further measurement for diabetes, and the performance at study (or population) level assesses how well the prediction equation works for diabetes surveillance. Most of the prediction equations had acceptable performance for estimating the probability that a person without diagnosed diabetes at a specific level of one glycemic biomarker (FPG or HbA1c) was above the clinical threshold for the other (Extended Data Tables 3 and 4). Specifically, the C-statistic ranged 0.85–0.90 for prediction equations that used either biomarker to predict the elevated level of the other. The mean errors were between −0.18 and −0.65 percentage points and the mean absolute errors were between 2.32 and 3.30 percentage points. The best-performing models for predicting whether participants had HbA1c ≥ 6.5% using FPG measurement included BMI and region-specific terms for FPG, referred to as models 5 and 8 in Extended Data Tables 2 and 3. These two models had similar C-statistic. Model 5 had the smallest deviation and model 8 had the smallest bias. The addition of sex interaction terms did not improve model performance. The best models for predicting whether participants had FPG ≥ 7.0 mmol l^−1^ using HbA1c measurement were also models 5 and 8 (Extended Data Tables 2 and 4). The coefficients of these models are shown in Extended Data Tables 5 and 6.

**Fig. 3:**
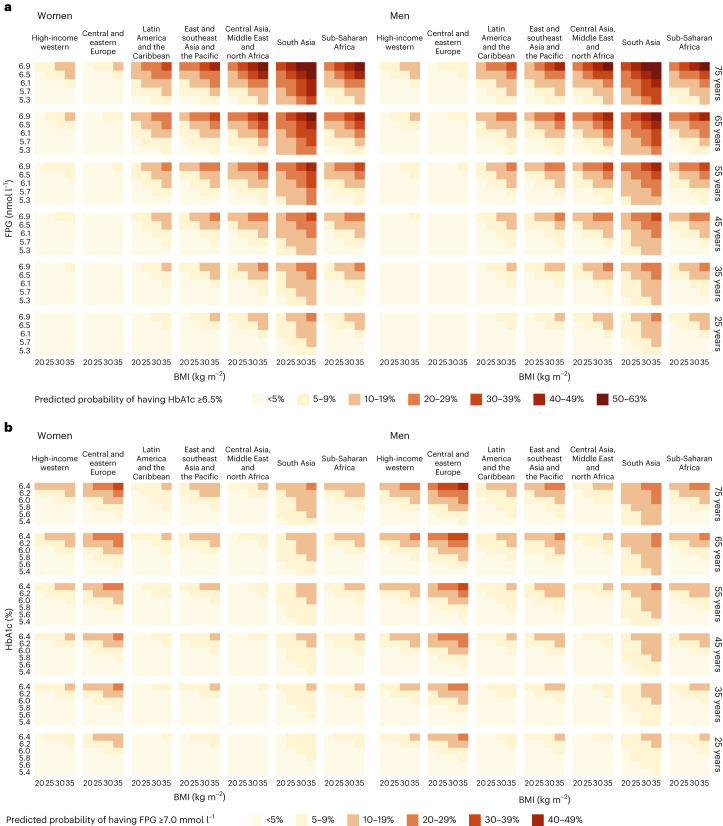
The predicted probability of having screen-detected diabetes with isolated elevated HbA1c or FPG. **a**,**b**, The probability, by sex, age and region, of participants who did not have previous diagnosis of diabetes of having elevated HbA1c (≥6.5%) at different FPG and BMI levels (**a**) and elevated FPG (≥7.0 mmol l^−1^) at different HbA1c and BMI levels (**b**). The probabilities were calculated using coefficients of prediction equation model 8, with measurement method set to laboratory for prediction. These results show the probability of having screen-detected diabetes if the second biomarker had been measured, for a person whose first biomarker was below the clinical threshold for diabetes diagnosis.

In Fig. 3, the coefficients from model 8 were used to calculate the probability that a person without a history of diabetes diagnosis, based on measurement of a single glycemic biomarker that is below the clinical threshold, would have elevated level of the other (elevated HbA1c at a specific FPG and BMI level (Fig. 3a) or elevated FPG at a specific HbA1c and BMI level (Fig. 3b)). For example, in south Asia, people aged 55 years and older, without a previous diabetes diagnosis, with obesity (BMI ≥ 30 kg m^−2^), whose FPG is 6.5–6.9 mmol l^−1^ have a 29–63% probability of having elevated HbA1c. In contrast, the probability of having elevated HbA1c remained no higher than 17% for men and women of the same age and FPG level in the high-income western region and central and eastern Europe, which means that screen-detected diabetes that is manifested as isolated elevated HbA1c is relatively rare in these two regions. For those whose HbA1c was measured, the probability of having elevated FPG was below 30% in every region except central and eastern Europe; the probability surpassed 20% only in those with high BMI and HbA1c levels.

**Fig. 4:**
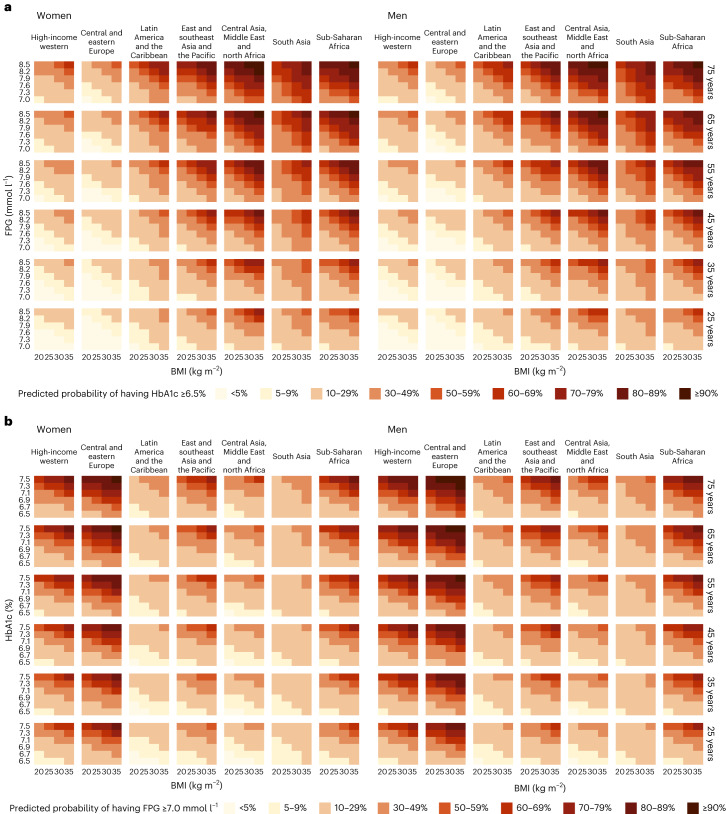
The predicted probability of having screen-detected diabetes with elevated levels of both FPG and HbA1c. **a**,**b**, The probability by sex, age and region of participants who did not have a previous diagnosis of diabetes of having elevated HbA1c (≥6.5%) at different FPG and BMI levels (**a**) and elevated FPG (≥7.0 mmol l^−1^) at different HbA1c and BMI levels (**b**). The probabilities were calculated using coefficients of prediction equation model 8, with measurement method set to laboratory for prediction. These results show the probability that the second biomarker, had it been measured, would be above the clinical threshold for diabetes diagnosis, for a person whose first biomarker was above the clinical threshold for diabetes diagnosis. Having elevated levels of both biomarkers has high positive predictive value for subsequent clinical diagnosis and risk of complications^14,47^.

In Fig. 4, the coefficients from model 8 were used to calculate the probability that a person without a history of diabetes diagnosis, based on measurement of a single glycemic biomarker that is above the clinical threshold, would have elevated level of the other (elevated HbA1c at a specific FPG and BMI level (Fig. 4a) or elevated FPG at a specific HbA1c and BMI level (Fig. 4b)). These results show that people without a previous diagnosis who had an elevated level of one diabetes biomarker had varying probabilities of also being elevated for the other depending on region, age, sex and BMI. In particular, for those with screen-detected elevated HbA1c, the probability of also having FPG ≥ 7.0 mmol l^−1^ surpassed 90% in some region-age-BMI combinations. The exceptions were south Asia and Latin America and the Caribbean, where isolated elevated HbA1c and isolated elevated FPG were both common and hence only partially predicted one another.

## Discussion

Our analysis of pooled global data showed that the use of either FPG or HbA1c alone might substantially underestimate the burden of diabetes relative to the number of people who would have elevated levels of either glycemic measure, especially in low- and middle-income countries where diagnosis rates are currently low. We also presented prediction equations to help allocate finite resources for measurement of HbA1c in settings where FPG (but not HbA1c) is routinely measured due to logistic or cost constraints. The prediction equations can also be used to enhance diabetes surveillance, to adjust the estimated prevalence in the majority of population-based health surveys which measure only one biomarker.

Our results, based on a large number of studies from different regions of the world, are consistent with a previous smaller study with data from mostly high-income western countries^43^ and with the collective results from studies done in individual countries^22–42^ in identifying substantial variation in diabetes classified by FPG versus HbA1c across regions. None of the previous studies had sufficient geographical coverage or participants to robustly quantify regional differences in how those with previously undiagnosed diabetes that were identified based on elevation of FPG and HbA1c, in isolation or together, as we did. A study using baseline data from the ORIGIN trial^48^, which covered people with diabetes or prediabetes from 40 countries, did not quantify the concordance and discordance of diabetes based on different biomarkers but its graphical results indicated smaller differences in FPG-HbA1c relationship between Europe and north America than between these regions and Asia or south America. We found that sex, age and BMI were predictors of having concordant versus discordant elevated FPG and elevated HbA1c, which is consistent with results from studies in individual countries^22,32,34,40,49^. Finally, to our knowledge, our prediction equations are the only global and generalizable tool for predicting the probability of being classified as having diabetes based on one glycemic biomarker based on measurement of another. A previous regression related HbA1c to average glucose^50^ (but not fasting glucose). This relationship is currently used by the American Diabetes Association for assessing glycemic control^51^ and not for inferring new diagnosis of diabetes. It used data from only 507 individuals, 422 of whom were non-Hispanic White. The data came from ten centers, of which nine were in the United States and Europe. Over half (268) had type 1 diabetes, which is the less common form of diabetes in adults. The conversions did not account for other traits such as BMI and age, nor was the performance of the prediction equation validated in data that were not used in its derivation.

The strengths of our study include the amount, quality and geographical diversity of data, with studies from seven of eight major world regions. We carefully checked that data on biomarkers of diabetes and previous diagnosis were of high quality and consistent across studies as stated in detail in the Methods. The scale, quality and consistency of data allowed the characterization of the relationship between these glycemic biomarkers and the development of prediction equations that can inform the allocation of resources toward closing the global diagnosis and monitoring gaps.

Our study is also affected by limitations that apply to data pooling analyses, especially those that use data collected in different countries and time periods. Despite our extensive efforts to identify and access data, we had limited data in some regions and none from Pacific island nations in the Oceania region. We did not analyze concordance and discordance with OGTT because few studies, mostly from high-income countries, had data on all three glycemic biomarkers and because the use of OGTT in clinical settings is largely for diagnosis of gestational diabetes and not for population surveillance. The use of OGTT would identify additional people as having diabetes above and beyond those identified with FPG and HbA1c^25,28^. We did not analyze time trends of diagnosed and screen-detected diabetes, which should be the subject of future work, as conducted for hypertension^52^. Although we checked all data sources and their characteristics thoroughly, and accounted for whether a study had measured FPG and HbA1c in a laboratory or using a portable device, other unobserved differences might remain due to differing methods. Examples include differences in assays used for measuring FPG and HbA1c. We attempted to mitigate these differences by limiting our data to studies with mid-year of 2000 and later, a period over which HbA1c assays were more likely to be standardized, and by including the study-level random effects in our models, which remove the influence of unobserved differences across studies. Beyond our finding that the results were not sensitive to exclusion of studies that used a portable device (Extended Data Table 7), studies that have tested different devices on the same set of samples have found high correlations (>0.97) among their measurements and between these devices and reference laboratory methods^53,54^. We did not have consistent data from all studies on other potential determinants of concordant versus discordant elevated levels of FPG and HbA1c, such as genetics, fasting duration, time between puncture and centrifuge, measures of insulin resistance and pre-existing disease status and comorbidities (for example, liver disease, hemoglobinopathies and anemia) that might have differential influence on FPG and HbA1c. These variables should ideally be the subject of coordinated multicenter studies with consistent data collection methods in different regions and populations; however, such studies would be very costly especially as the number of outcomes and variables increases. There is intraindividual variation in FPG, and to a lesser extent HbA1c^55^, which could reduce the concordance between FPG and HbA1c, and repeated measurements of FPG may improve its concordance with HbA1c^39^. Finally, while the studies that were used to define the diagnostic cutoff points were all based on single measurements of glycemia^8,56^, as are epidemiological and surveillance studies^44,57–59^, many clinical guidelines recommend using a second confirmatory test for diabetes diagnosis and initiating treatment^10–13^ (we note that there is variation in this guidance, for example while the American Diabetes Association requires two above-threshold tests for diagnosing diabetes in most cases^10^, the European Association for the Study of Diabetes only advises doing so^11^, the World Health Organization only recommends repeated testing for asymptomatic patients^13^, and the International Diabetes Federation further limits repeated testing to when the first measurement is close to the threshold for diagnosis^12^). A key reason for clinical guidelines recommending a confirmatory test is to minimize risks of erroneous results, for example, due to mis-recording of laboratory results or large intraindividual variability (which is more relevant for FPG than HbA1c), potentially leading to a lifelong (mis-)diagnosis for an individual patient. This is not a relevant issue in prevalence studies in a population, as random measurement error and fluctuations in one direction are approximately balanced by those in the opposite direction. Reflecting the difference between the clinical and epidemiological approaches to diabetes definition, we referred to those without a previous diagnosis who had biomarker levels above the clinical thresholds as screen-detected diagnosis, and our prediction equations should be considered a tool for triaging some people at specific levels of FPG for measurement of HbA1c, and possibly vice versa, rather than a tool for conferring a diagnosis.

The observed variation in the composition of screen-detected diabetes across regions may be due to a number of factors. Some genetic and phenotypic factors that affect fasting glucose and glucose metabolism through their effects on β-cell function and insulin sensitivity may be more common in some regions or ethnic groups^60–64^. Other non-glycemic factors, including anemia due to iron deficiency or malaria, certain hemoglobin variants (for example, HbS and HbF), other hemoglobinopathies, polycythemia due to living in high altitude, liver and kidney diseases, HIV and certain drugs such as antiretroviral therapy for HIV, can also affect HbA1c and FPG differently^65–77^. Some of these factors, including malaria-induced and iron deficiency anemia, hemoglobinopathies such as sickle cell disease and thalassemia, and antiretroviral therapy, are more prevalent in parts of Asia and Africa^78–80^, and may have shifted the population distribution of HbA1c or affected its measurement^77^. One study from South Africa found that the impact of these factors on HbA1c were small^81^. Guidelines recommend the use of a glucose test for diabetes diagnosis in those with such conditions^10^. Smoking and alcohol use, which vary geographically, may differentially affect HbA1c and FPG^82,83^. Finally, the composition of diabetes that was detected through screening in the survey depends on whether those with a previous diagnosis were identified based on FPG or HbA1c. For example, with increasing use of HbA1c in clinical settings in high-income countries^84^, a smaller proportion of people with screen-detected diabetes would have elevated HbA1c.

Although both FPG and HbA1c are associated with increased risk of microvascular and macrovascular complications^2,85,86^, the current evidence on the health implications of having discordant versus concordant elevation of FPG and HbA1c is limited. The few available studies found worse outcomes on the health risks associated with concordant elevation of FPG and HbA1c than discordant elevation, but had mixed findings about how isolated elevation of the two biomarkers compare^39,87,88^. To the extent that both FPG and HbA1c are predictors of risk of complications and mortality, reliance on a single biomarker may miss or delay diagnosis of diabetes in some people and hence increase their risk of complications. This issue is especially relevant in low- and middle-income countries where resource constraints make FPG the more common approach to diagnosis, possibly because the measurement of HbA1c requires equipment or reagents that are more costly or because standardization of the HbA1c laboratory process requires specialist training that is not as widely available^89–93^. With finite resources, our prediction equations can help to triage some people for the measurement of a second biomarker, often HbA1c, and enhance early detection of diabetes and close the global diagnosis shortfall^14^. For surveillance, the use of a single biomarker, so far largely FPG^44–46^, underestimates the burden of diabetes and does so to a larger extent in low- and middle-income countries where a larger share of conditions such as diabetes (and hypertension^52^) remains undiagnosed. Our prediction equations can help provide a more complete picture of the burden of diabetes in different regions.

## Methods

The pooled analysis was approved by Imperial College London Research Ethics Committee and complies with all relevant ethical regulations. The participating studies followed their institutional approval process at the time of data collection.

### Data

We used data collated by the NCD-RisC. The data sources included national and multi-country measurement surveys that were either publicly available or identified and accessed through contacts with relevant government or academic partners. Additionally, we searched and reviewed published studies as detailed previously^44^ and invited eligible studies to join NCD-RisC, as we did with participating studies in previous pooled analyses of cardiometabolic risk factors^96–99^. The NCD-RisC database is continuously updated through the above routes and through periodic requests to NCD-RisC members to suggest additional sources in their countries.

The inclusion criteria for this analysis were (1) data were collected using a probabilistic sampling method with a defined sampling frame; (2) data were from population samples at the national, subnational (defined as covering one or more subnational regions, more than three urban communities or more than five rural communities) or community level (defined as having up to three urban communities or up to five rural communities); and (3) both FPG and HbA1c were measured. Studies were excluded if they had (1) enrolled participants based on health status or cardiovascular risk; (2) were conducted only among ethnic minorities or specific educational, occupational or other socioeconomic subgroups; (3) recruited participants through health facilities, except studies based on the primary care system in high-income and central European countries with universal insurance; (4) had not measured either FPG or HbA1c; (5) had not instructed participants to fast for at least 6 h before FPG measurement; (6) had only measured FPG or HbA1c in the subset of participants who had known diabetes; (7) had measured HbA1c only in a subset of participants selected based on their levels of FPG and vice versa; (8) had not collected information on a previous diagnosis of diabetes; and (9) their mid-year was before 2000, before HbA1c assays were widely standardized^100^.

At least two independent people ascertained that each data source met the inclusion criteria. All NCD-RisC members were asked to review the list of data sources from their country, to verify that the included data met the inclusion criteria and were not duplicates. When FPG and/or HbA1c data were missing for more than 10% of participants in a survey, we checked the study design documentation to verify missingness at random so that the above inclusion criteria were met. Questions and clarifications were discussed with NCD-RisC members and resolved before data were incorporated in the database. For each data source, we recorded the study population, sampling approach, years of measurement and measurement methods, including whether FPG and HbA1c were measured in a laboratory or using a portable point-of-care device. In 11 studies, fasting glucose was measured in capillary whole blood; four of these used equipment that reported plasma-equivalent values. We converted the measurements from the other seven studies to plasma-equivalent using the relationship in a study that compared different types of specimens^101^. In a sensitivity analysis, we excluded these 11 studies from the analysis.

We established whether a participant had diagnosed diabetes using questions worded as variations of ‘Have you ever been told by a doctor or other health professional that you had diabetes, also called high blood sugar?’ In some surveys, the question on previous diabetes diagnosis was asked only if a participant had answered ‘yes’ to an earlier question, usually worded as ‘Have you ever been screened for diabetes?’ or ‘Have you ever had your blood glucose measured?’. In these cases, participants who answered ‘no’ to the first question were coded as not having been diagnosed with diabetes. We also considered participants who used diabetes medication such as metformin or insulin as having diabetes. Survey data typically do not separate type 1 and type 2 diabetes in adults, but studies that had data on these subtypes show that most (85–95%) cases of diabetes in adults are type 2 diabetes^102^.

The data cleaning and use process is summarized in Fig. 1 and the list of data sources and their characteristics are stated in Supplementary Table 1.

### Statistical analysis

We divided the participants into those who had a previous diagnosis of diabetes (hereafter referred to as diagnosed diabetes), those without a previous diagnosis of diabetes who had elevated FPG (FPG ≥ 7.0 mmol l^−1^) and/or elevated HbA1c (HbA1c ≥ 6.5%) (referred to as screen-detected diabetes) and the remainder who did not have a previous diagnosis, elevated FPG, or elevated HbA1c. We conducted the following three analyses.

#### Screen-detected diabetes by FPG and HbA1c

We graphically presented how total diabetes is divided into diagnosed and screen-detected diabetes, and how screen-detected diabetes is further divided into those manifested as only elevated FPG (FPG ≥ 7.0 mmol l^−1^ and HbA1c < 6.5%, referred to as isolated elevated FPG), only elevated HbA1c (HbA1c ≥ 6.5% and FPG < 7.0 mmol l^−1^, referred to as isolated elevated HbA1c) or elevated levels of both FPG and HbA1c. We report crude and age-standardized prevalence. We calculated crude prevalence using data from all participants regardless of age. We calculated age-standardized prevalence as the weighted mean of the age-specific values using the World Health Organization standard population^103^. We also graphically described the relationship of FPG and HbA1c among people without diagnosed diabetes.

#### Association with individual and study characteristics

We fitted regression models to examine what individual and study-level factors were associated with whether participants with screen-detected diabetes were identified by elevated FPG, elevated HbA1c or elevated levels of both. We fitted three separate log-binomial regressions, with each of the three outcomes (isolated elevated FPG, isolated elevated HbA1c and elevated levels of both) as a distinct dependent variable. A log-binomial regression estimates the association of each independent variable with the probability of a participant falling in each of the three categories as PR. The individual level independent variables were sex, age and BMI; the study-level variables were region, study year, whether FPG and HbA1c were measured in a laboratory or using a portable device (to account for differences in measurement between them^53,54^) and percentage of participants with diabetes who had been diagnosed before in each study. The regressions also included a study-level random effect to account for unobserved factors that led to systematic differences in each study compared to others^104,105^.

We fitted the log-binomial regressions using Bayesian model fitting implemented in MultiBUGS (v.2.0)^106^. Bayesian model fitting has better estimation performance for log-binomial model than a frequentist approach^107^. We used a normal distribution with mean of zero and s.d. of 0.01 as the prior for the regression coefficients and a uniform distribution on 0.01–2.00 as the prior for the s.d. of study-level random effects. We ran four chains and assessed convergence visually using trace plots. After burn-in and thinning, we kept 50,000 draws to represent the posterior distributions of the PRs. We report PRs and their 95% CrIs as the mean and the 2.5th and 97.5th percentiles of their posterior distributions. We report the posterior probability that a PR with posterior mean estimate >1.0 is less than one and vice versa for PRs <1.0; the posterior probabilities are analogous to *P* values in a frequentist analysis.

#### Prediction equations

We tested nine logistic regression models for estimating the probability that a person without diagnosed diabetes at a specific level of FPG had an HbA1c over the clinical threshold for diabetes (HbA1c ≥ 6.5%). The variables in the models were selected based on clinical and epidemiological relevance and data availability. The variables included FPG as well as sex, age, BMI, glycemic measurement method (laboratory based or via a portable device) and region. The nine prediction models (Extended Data Table 2) differed by the predictors included and whether the coefficient of the FPG term was allowed to vary by sex and region. In all models, we included a study-level random effect to account for unobserved factors that led to systematic differences in each study compared to others^104,105^. We also tested the inclusion of nonlinear (square and cubic) terms of FPG, year of data collection and other interaction terms; these models performed worse than those without the additional terms as evaluated by the metrics below and are not presented. We did not interact age, which is a continuous variable, with FPG and other terms, to avoid overfitting. We fitted and evaluated all prediction models in R (v.4.2.1)^108^.

We assessed the performance of the models in predicting (1) individual participants’ status of having HbA1c ≥ 6.5% based on their FPG and (2) the prevalence of HbA1c ≥ 6.5% for an entire study. The performance at the individual level reflects how well the prediction equation works for triaging patients for further measurement for diabetes, and the performance at study (or population) level assesses how well it works for diabetes surveillance. We used the C-statistic to assess individual-level performance and mean error and mean absolute error between the predicted and observed prevalence for population-level performance. The C-statistic measures how well a prediction equation distinguishes individuals with higher risk from those with lower risk. Mean error assesses whether there is systematic difference (bias) in the predicted prevalence compared to the observed one and mean absolute error assesses any deviation of the predicted prevalence from the observed prevalence. We calculated error by study, sex and age group (18–39 years, 40–59 years and 60 years and older).

We evaluated the performance of the models in 20 rounds of tenfold cross-validation^109^. In each fold of each round, we held out all data from a random 10% of studies, fitted the model to the data from the remaining 90% of studies and made estimates for the held-out observations. We repeated this process ten times, each time holding out a different 10% of studies so that each study was held out exactly once. We calculated the above individual-level and population-level performance metrics for all held-out observations. We repeated the tenfold cross-validation 20 times and report the means and ranges of the performance metrics from all 20 rounds.

We repeated the same process for predicting the probability of having FPG ≥ 7.0 mmol l^−1^ based on HbA1c.

### Ethics and inclusion

This research followed the recommendations set out in the Global Code of Conduct for Research in Resource-Poor Settings.

### Reporting summary

Further information on research design is available in the Nature Portfolio Reporting Summary linked to this article.

## Online content

Any methods, additional references, Nature Portfolio reporting summaries, source data, extended data, supplementary information, acknowledgements, peer review information; details of author contributions and competing interests; and statements of data and code availability are available at 10.1038/s41591-023-02610-2.

### Supplementary information

Supplementary Information Supplementary Table 1.

Reporting Summary

## Figures and Tables

**Extended Data Fig. 1:**
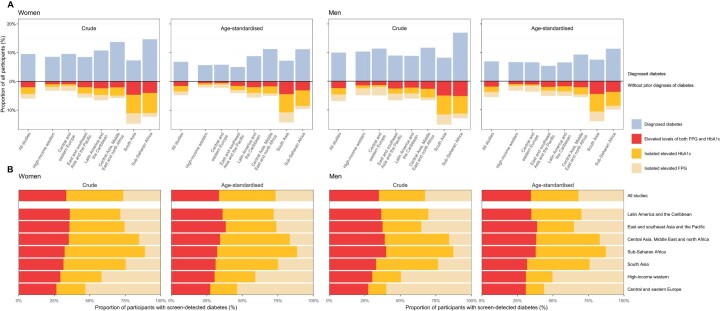
Extent and composition of diagnosed and screen-detected diabetes by region and sex. (**a**) Crude and age-standardized proportion of participants with diagnosed or screen-detected diabetes, and, for those without prior diagnosis, whether they had isolated elevated FPG (FPG ≥7.0 mmol/L and HbA1c < 6.5%), isolated elevated HbA1c (HbA1c ≥6.5% and FPG < 7.0 mmol/L) or elevated levels of both, and (**b**) crude and age-standardized proportion of participants with screen-detected diabetes who had isolated elevated FPG, isolated elevated HbA1c or elevated levels of both, by region and sex. Its contents are the same as the segment of Panel A that is below the zero line, scaled to 100% so that the composition of screen-detected diabetes can be compared across regions, regardless of its total prevalence. Having elevated levels of both biomarkers has high positive predictive value for subsequent clinical diagnosis and risk of complications^14,47^, and hence this group is similar to clinically-diagnosed diabetes. In panel A, regions are ordered by the total proportion of participants who had diagnosed and screen-detected diabetes. In panel B, regions are ordered by the crude proportion of participants with screen-detected diabetes who had elevated levels of both FPG and HbA1c.

**Extended Data Table 1:**
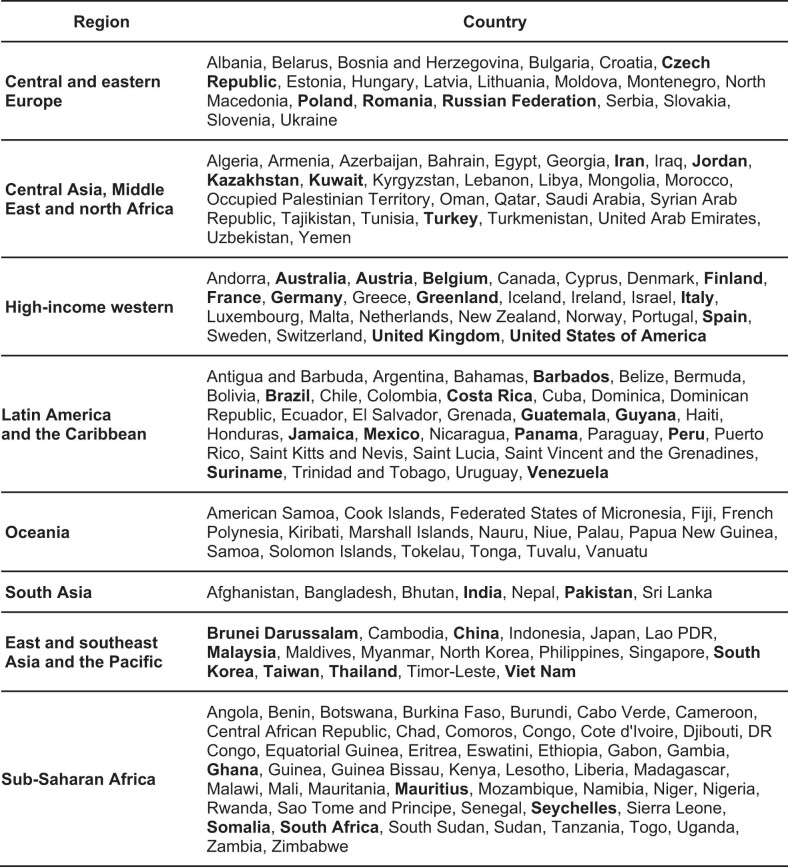
List of analysis regions and countries in each region. The data used in the analysis came from countries shown in bold

**Extended Data Table 2:**
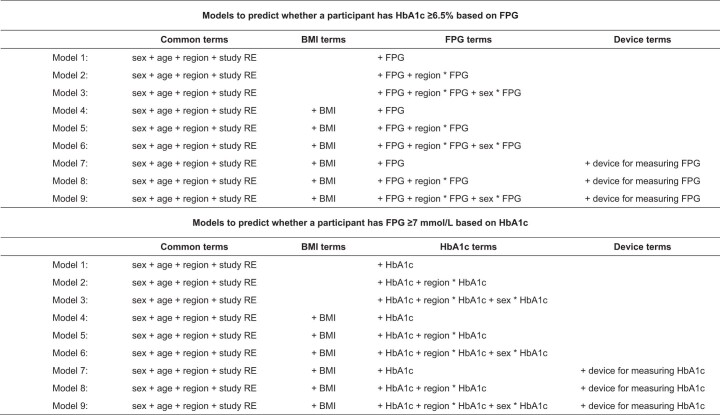
Specification of models tested to predict whether a participant has HbA1c ≥6.5% based on FPG levels, and to predict whether a participant has FPG ≥7.0 mmol/L based on HbA1c levels

* denotes statistical interaction.

Age, FPG, HbA1c and BMI were normalized using the following values (approximately equal to mean and standard deviation across all participants):

Age: centered at 50 years, divided by 15 years

FPG: centered at 5.5 mmol/L, divided by 1.0 mmol/L

HbA1c: centered at 5.5%, divided by 0.7%

BMI: centered at 26.5 kg/m^2^, divided by 5.0 kg/m^2^

FPG: fasting plasma glucose; BMI: body-mass index; RE: random effect.

**Extended Data Table 3:**
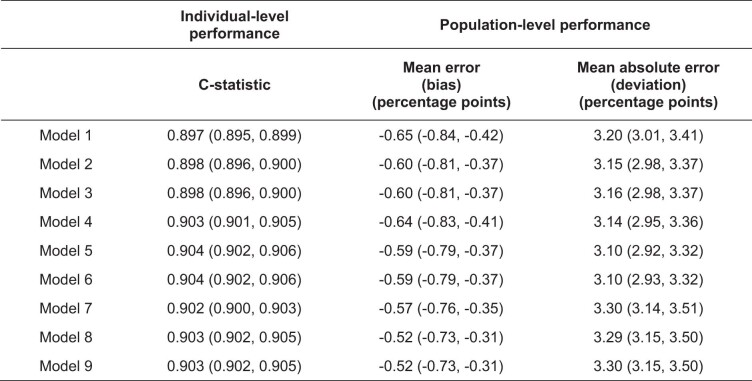
Performance of models for predicting whether a participant whose FPG was measured had HbA1c ≥6.5%

The reported values are the means and ranges over 20 rounds of 10-fold cross-validation. See Extended Data Table 2 for details of model specifications.

**Extended Data Table 4:**
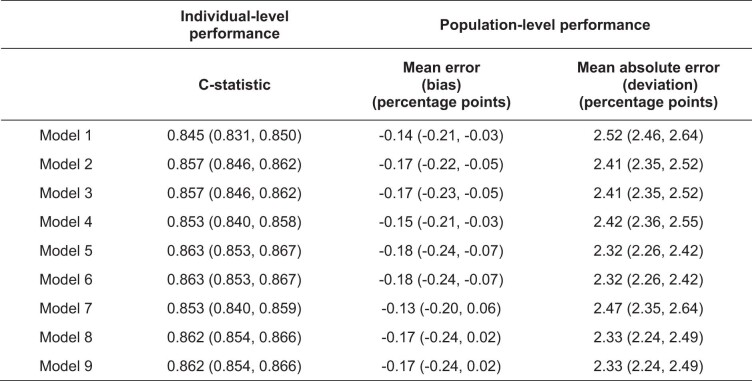
Performance of models for predicting whether a participant whose HbA1c was measured had FPG ≥7.0 mmol/L

The reported values are the means and ranges over 20 rounds of 10-fold cross-validation. See Extended Data Table 2 for details of model specifications.

**Extended Data Table 5:**
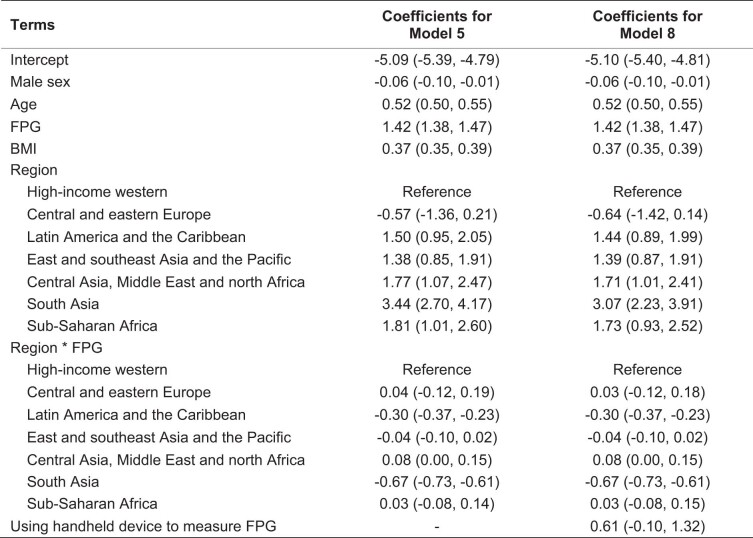
Coefficients of the best-performing prediction equations for whether a participant whose FPG was measured had HbA1c ≥6.5%

The reported coefficients are the means and 95% confidence intervals.

**Extended Data Table 6:**
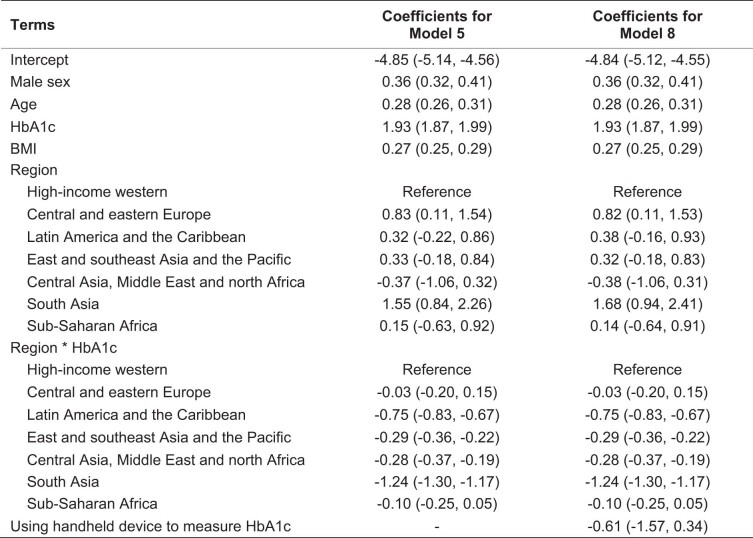
Coefficients of the best-performing prediction equations for whether a participant whose HbA1c was measured had FPG ≥7.0 mmol/L

The reported coefficients are the means and 95% confidence intervals.

**Extended Data Table 7:**
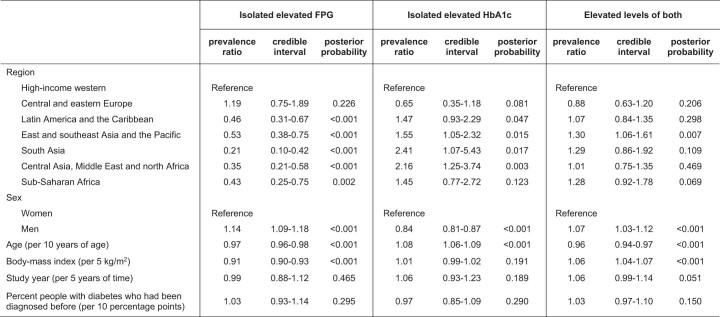
Association of whether screen-detected diabetes is presented as isolated elevated FPG, isolated elevated HbA1c or elevated levels of both with individual and study characteristics, excluding studies that had measured FPG using a portable device

The association with each variable is reported as prevalence ratios, adjusted for all other variables in the table, in the regressions described in Methods in which data from individual participants with screen-detected diabetes were used.

## Data Availability

Data used in this research are governed by data-sharing protocols of participating studies. Contact information for data providers can be obtained from www.ncdrisc.org and 10.5281/zenodo.8169145.

## Code availability

The computer code for the log-binomial regression in this work is available at www.ncdrisc.org and 10.5281/zenodo.8169145.
