# Supplementary material for: Global variation in diabetes diagnosis and prevalence based on fasting glucose and hemoglobin A1c
Source: Nat Med. 2023 Nov 9;29(11):2885–901. doi: 10.1038/s41591-023-02610-2 (PMC10667106; doi:10.1038/s41591-023-02610-2)
Supplement: Supplementary file 1 — Supplementary Table 1. [file 41591_2023_2610_MOESM1_ESM.pdf]

nature medicine

Article https://doi.org/10.1038/s41591-023-02610-2

Global variation in diabetes diagnosis and prevalence based on fasting glucose and hemoglobin A1c

In the format provided by the authors and unedited

Supplementary Table 1. List of data sources and their characteristics.

	Country	Data years	Survey/Study name/Citation	Level of representative-ness	Age range used in the analysis		Sample size used in the analysis		Sample size used in the analysis with data on fasting glucose and HbA1c		Whether glucose was measured in a laboratory or by a POC portable device	Whether HbA1c was measured in a laboratory or by a POC portable device	Region	Notes
					Female	Male	Female	Male	Female	Male				
1	Australia	1999-2000	The Australian Diabetes, Obesity and Lifestyle Study (AusDiab)	National	25+	25+	6,140	5,048	6,103	5,013	Lab	Lab	High-income western	
2	Australia	1999-2003	North West Adelaide Health Study	Community	18+	18+	2,117	1,924	2,089	1,891	Lab	Lab	High-income western	
3	Austria	2010-2012	Austrian Study on Nutritional Status 2012	National	18-80	18-80	283	184	267	174	Lab	Lab	High-income western	
4	Barbados	2011-2013	Health of the Nation (HotN)	National	25+	25+	741	469	634	396	Lab	Portable	Latin America and the Caribbean	
5	Belgium	2018-2019	Belgian Health Examination Survey	National	18+	18+	617	560	521	483	Lab	Lab	High-income western	
6	Brazil	2010-2015	Baependi Heart Study	Community	18+	18+	1,409	1,059	1,166	814	Lab	Lab	Latin America and the Caribbean	
7	Brazil	2014-2015	EpiFloripa Adults Cohort Study	Community	25-65	25-65	497	366	410	298	Lab	Lab	Latin America and the Caribbean	
8	Brunei Darussalam	2010-2011	National Health And Nutritional Status Survey (NHANSS)	National	20-75	20-75	798	668	742	613	Lab	Lab	East and southast Asia and the Pacific	
9	China	2009	China Health and Nutrition Study	National	18+	18+	5,165	4,814	4,502	3,997	Lab	Lab	East and southast Asia and the Pacific	1
10	China	2011-2012	China Health and Retirement Longitudinal Study (CHARLS), baseline survey	National	45+	45+	7,085	6,426	4,675	4,059	Lab	Lab	East and southast Asia and the Pacific	
11	Costa Rica	2004-2006	Costa Rican Longevity and Healthy Aging Study Pre-1945 Cohort Wave 1	National	60+	60+	1,529	1,283	1,415	1,174	Lab	Lab	Latin America and the Caribbean	
12	Czech Republic	2002-2005	Health, Alcohol and Psychosocial Factors In Eastern Europe (HAPIEE)	Subnational	45-70	45-70	4,675	4,068	432	414	Lab	Lab	Central and eastern Europe	
13	Czech Republic	2015-2018	Czech post-MONICA	National	25-64	25-64	1,343	1,222	1,324	1,208	Lab	Lab	Central and eastern Europe	
14	Finland	2000-2001	Health 2000 Survey	National	30+	30+	4,035	3,308	2,210	1,861	Lab	Lab	High-income western	
15	Finland	2011	Young Finns Study 2011 (rural)	National	34-49	34-49	435	367	420	363	Lab	Lab	High-income western	
16	Finland	2011	Young Finns Study 2011 (urban)	National	34-49	34-49	646	514	624	495	Lab	Lab	High-income western	
17	Finland	2011-2012	Health 2011 Survey	National	30+	30+	542	448	146	120	Lab	Lab	High-income western	
18	Finland	2012	Northern Finland Birth Cohort 1966	Community	45-47	45-47	2,804	2,189	2,430	1,871	Lab	Lab	High-income western	
19	Finland	2017	The FinHealth Survey	National	18+	18+	3,637	3,231	1,438	1,326	Lab	Lab	High-income western	
20	Finland	2018-2019	Savitaipale Study, 22-year Follow-up	Community	62-86	62-86	347	268	339	262	Lab	Lab	High-income western	
21	Finland	2019-2020	Northern Finland Birth Cohort 1986	Community	33-35	33-35	875	530	873	529	Lab	Lab	High-income western	
22	France	2008-2010	The Three City Study	Community	73+	73+	2,984	1,650	443	249	Lab	Lab	High-income western	
23	Germany	2000-2002	Epidemiological study of the chances of prevention, early recognition and optimal treatment of chronic diseases in an elderly population (ESTHER)	Subnational	50-75	50-75	5,321	4,365	4,393	3,541	Lab	Lab	High-income western	
24	Germany	2000-2003	Heinz Nixdorf Recall Study	Subnational	45-75	45-75	2,416	2,394	1,551	1,564	Lab	Lab	High-income western	2
25	Germany	2008-2012	Study of Health in Pomerania, second cohort (SHIP-TREND)	Subnational	20-79	20-79	2,228	2,096	1,462	1,242	Lab	Lab	High-income western	
26	Ghana	2012-2014	Research on Obesity and Diabetes among African Migrants (RODAM), control group (rural)	Subnational	25+	25+	634	407	453	273	Lab	Lab	Sub-Saharan Africa	
27	Ghana	2012-2014	Research on Obesity and Diabetes among African Migrants (RODAM), control group (urban)	Subnational	25+	25+	1,007	394	975	386	Lab	Lab	Sub-Saharan Africa	
28	Greenland	2005-2010	Population Health Survey in Greenland	National	18+	18+	1,726	1,351	1,706	1,330	Lab	Lab	High-income western	
29	Greenland	2016-2019	Population Health Survey in Greenland	National	25+	25+	1,046	833	763	573	Lab	Lab	High-income western	
30	Guatemala	2015-2017	Nutrition on early childhood and metabolomic and cardiometabolic profile on adulthood (META)	Community	37-55	37-55	310	213	302	207	Lab	Lab	Latin America and the Caribbean	
31	Guyana	2016	STEPS	National	18-69	18-69	1,585	1,068	405	238	Portable	Lab	Latin America and the Caribbean	
32	India	2001-2004	Chennai Urban Rural Epidemiology Study	Community	20+	20+	1,254	1,096	1,238	1,090	Lab	Lab	South Asia	
33	India	2007-2008	Urban population in Hyderabad	Community	20-60	20-60	1,552	1,512	58	66	Portable	Portable	South Asia	
34	India	2008-2010	ICMR-India Diabetes (INDIAB) Study, Phase I	National	20+	20+	6,401	6,457	1,128	1,328	Portable	Lab	South Asia	
35	India	2010-2012	Centre for cArdiometabolic Risk Reduction in South-Asia (CARRS) - Surveillance Study	Community	20+	20+	6,403	5,867	5,417	4,704	Lab	Lab	South Asia	3
36	India	2012-2013	ICMR-India Diabetes (INDIAB) Study, Phase II	Subnational	20+	20+	10,439	7,859	2,149	2,173	Portable	Lab	South Asia	
37	India	2012-2015	ICMR-India Diabetes (INDIAB) Study, North East Phase	Subnational	20+	20+	12,281	10,032	2,275	2,566	Portable	Lab	South Asia	
38	India	2014-2015	Control of Hypertension In Rural India (CHIRI) - Rishi Valley	Community	18+	18+	3,507	2,581	999	686	Portable	Portable	South Asia	
39	Iran	2013-2014	Gilan Eye Study	Subnational	50+	50+	1,472	1,095	1,159	840	Lab	Lab	Central Asia, Middle East and north Africa	
40	Iran	2016	Iran STEPS 2016	National	25+	25+	13,843	13,038	10,017	8,704	Lab	Lab	Central Asia, Middle East and north Africa	
41	Iran	2020-2021	STEPS	National	25+	25+	13,979	11,202	10,212	7,770	Lab	Lab	Central Asia, Middle East and north Africa	
42	Italy	2000	Bruneck Study	Community	50-89	50-89	361	331	361	331	Lab	Lab	High-income western	
43	Jamaica	2016-2017	Jamaica Health and Lifestyle Survey	National	18+	18+	1,635	1,028	998	574	Portable	Portable	Latin America and the Caribbean	
44	Jordan	2016-2017	National Cardiovascular Diseases and Diabetes Study (NCDDS)	National	18+	18+	2,763	1,182	2,696	1,166	Portable	Lab	Central Asia, Middle East and north Africa	
45	Kazakhstan	2019	A health status assessment of a population of Karaganda urban region	Community	18+	18+	672	326	664	316	Lab	Lab	Central Asia, Middle East and north Africa	

	Country	Data years	Survey/Study name/Citation	Level of representative-ness	Age range used in the analysis		Sample size used in the analysis		Sample size used in the analysis with data on fasting glucose and HbA1c		Whether glucose was measured in a laboratory or by a POC portable device	Whether HbA1c was measured in a laboratory or by a POC portable device	Region	Notes
					Female	Male	Female	Male	Female	Male				
46	Kazakhstan	2021	Prevalence of NCD Risk Factors in Kazakhstan	Subnational	18-69	18-69	814	802	813	802	Lab	Lab	Central Asia, Middle East and north Africa	4
47	Kuwait	2008-2009	National Nutrition Program for the State of Kuwait	National	18-86	18-86	530	459	475	412	Lab	Lab	Central Asia, Middle East and north Africa	
48	Kuwait	2011-2014	Kuwait Diabetes Epidemiology Program	National	18-82	18-82	2,143	2,783	2,137	2,776	Lab	Lab	Central Asia, Middle East and north Africa	
49	Kuwait	2014	STEPS	National	18-69	18-69	2,405	1,456	1,574	955	Lab	Lab	Central Asia, Middle East and north Africa	
50	Malaysia	2008	Metabolic Syndrome Study in Malaysia (rural)	National	18+	18+	1,368	754	1,253	686	Lab	Lab	East and southast Asia and the Pacific	
51	Malaysia	2008	Metabolic Syndrome Study in Malaysia (urban)	National	18+	18+	1,449	769	1,397	733	Lab	Lab	East and southast Asia and the Pacific	
52	Mauritius	2015	Mauritius Noncommunicable Disease Survey	National	20-74	20-74	1,954	1,626	1,952	1,622	Lab	Lab	Sub-Saharan Africa	
53	Mauritius	2015	Mauritius Noncommunicable Disease Survey 1998 Follow Up	National	20+	20+	1,182	887	1,179	886	Lab	Lab	Sub-Saharan Africa	
54	Mexico	2016	Encuesta Nacional de Salud y Nutricion	National	20+	20+	5,477	2,865	2,437	1,314	Lab	Lab	Latin America and the Caribbean	
55	Mexico	2018-2019	Encuesta Nacional de Salud y Nutricion	National	20+	20+	9,798	7,584	7,311	5,418	Lab	Lab	Latin America and the Caribbean	
56	Mexico	2021	Encuesta Nacional de Salud y Nutricion	National	20+	20+	5,243	2,947	1,304	748	Lab	Lab	Latin America and the Caribbean	
57	Pakistan	2016-2017	National Diabetes Survey of Pakistan	National	20+	20+	5,715	4,448	4,261	3,273	Lab	Lab	South Asia	
58	Panama	2010-2011	Prevalencia de factores de riesgo asociados a enfermedad cardiovascular	Subnational	18+	18+	2,478	1,074	2,477	1,073	Lab	Lab	Latin America and the Caribbean	
59	Panama	2019	Encuesta Nacional de Salud de Panama (ENSPA)	National	18+	18+	12,014	4,846	3,754	1,110	Lab	Lab	Latin America and the Caribbean	
60	Peru	2007-2008	PERU MIGRANT Study	Community	30+	30+	522	466	521	466	Lab	Lab	Latin America and the Caribbean	
61	Peru	2009-2012	CRONICAS Cohort Study	Subnational	35+	35+	1,849	1,740	1,596	1,518	Lab	Lab	Latin America and the Caribbean	
62	Peru	2016-2017	Sceening of T2DM	Community	30-70	30-70	810	799	810	799	Lab	Lab	Latin America and the Caribbean	
63	Poland	2002-2005	Health, Alcohol and Psychosocial Factors In Eastern Europe (HAPIEE)	Community	45-70	45-70	5,485	5,220	469	478	Lab	Lab	Central and eastern Europe	
64	Poland	2011	NATPOL	National	18-79	18-79	1,209	1,143	1,066	991	Lab	Lab	Central and eastern Europe	
65	Poland	2015-2016	LIPIDOGRAM2015 & LIPIDOGEN2015 Study - National epidemiological study of lipid disorders and selected risk factors of cardiovascular disease in primary health care in Poland	National	18+	18+	8,690	5,034	1,075	713	Portable	Lab	Central and eastern Europe	
66	Romania	2011-2012	Study for the Evaluation of Prevalence of Hypertension and cArdiovascular Risk among the Adult Population of Romania - SEPHAR II	National	18-80	18-80	1,038	932	1,034	928	Lab	Lab	Central and eastern Europe	
67	Romania	2015-2016	Study for the Evaluation of Prevalence of Hypertension and cArdiovascular Risk among the Adult Population of Romania - SEPHAR III	National	18-80	18-80	1,034	936	1,033	935	Lab	Lab	Central and eastern Europe	
68	Russian Federation	2002-2005	Health, Alcohol and Psychosocial Factors In Eastern Europe (HAPIEE)	Community	45-70	45-70	5,079	4,244	480	720	Lab	Lab	Central and eastern Europe	
69	Seychelles	2013-2014	Seychelles Heart Survey IV	National	25-64	25-64	699	531	686	526	Portable	Lab	Sub-Saharan Africa	
70	Somalia	2016	The prevalence of selected risk factors for non-communicable diseases in Hargeisa, Somaliland: a cross-sectional study	Community	20-69	20-69	955	145	510	57	Lab	Lab	Sub-Saharan Africa	
71	South Africa	2008-2009	Cape Town Bellville South Cohort Study - Baseline evaluation I	Community	35-65	35-65	494	140	493	138	Lab	Lab	Sub-Saharan Africa	
72	South Korea	2011	Korea National Health and Nutrition Examination Survey	National	18+	18+	3,479	2,599	3,270	2,464	Lab	Lab	East and southast Asia and the Pacific	
73	South Korea	2012	Korea National Health and Nutrition Examination Survey	National	18+	18+	3,301	2,349	3,038	2,215	Lab	Lab	East and southast Asia and the Pacific	
74	South Korea	2013	Korea National Health and Nutrition Examination Survey	National	18+	18+	3,115	2,303	2,764	2,136	Lab	Lab	East and southast Asia and the Pacific	
75	South Korea	2014	Korea National Health and Nutrition Examination Survey	National	18+	18+	2,943	2,115	2,591	1,909	Lab	Lab	East and southast Asia and the Pacific	
76	South Korea	2015	Korea National Health and Nutrition Examination Survey	National	18+	18+	2,963	2,308	2,685	2,135	Lab	Lab	East and southast Asia and the Pacific	
77	South Korea	2016	Korea National Health and Nutrition Examination Survey	National	18+	18+	3,473	2,682	3,216	2,529	Lab	Lab	East and southast Asia and the Pacific	
78	South Korea	2017	Korea National Health and Nutrition Examination Survey	National	18+	18+	3,438	2,780	3,183	2,623	Lab	Lab	East and southast Asia and the Pacific	
79	South Korea	2018	Korea National Health and Nutrition Examination Survey	National	18+	18+	3,521	2,763	3,321	2,625	Lab	Lab	East and southast Asia and the Pacific	
80	South Korea	2019	Korea National Health and Nutrition Examination Survey	National	18+	18+	3,504	2,835	3,333	2,679	Lab	Lab	East and southast Asia and the Pacific	
81	South Korea	2020	Korea National Health and Nutrition Examination Survey	National	18+	18+	3,283	2,685	3,128	2,563	Lab	Lab	East and southast Asia and the Pacific	
82	Spain	2001-2003	DIabetes, Nutricion y Obesidad en la poblacion adulta de la Region de Murcia (DINO)	Subnational	20+	20+	837	719	834	715	Lab	Lab	High-income western	
83	Spain	2004	Vioque J et al., Obesity 16(3):664-70, 2008	Community	24+	24+	117	85	90	64	Portable	Portable	High-income western	

	Country	Data years	Survey/Study name/Citation	Level of representative-ness	Age range used in the analysis		Sample size used in the analysis		Sample size used in the analysis with data on fasting glucose and HbA1c		Whether glucose was measured in a laboratory or by a POC portable device	Whether HbA1c was measured in a laboratory or by a POC portable device	Region	Notes
					Female	Male	Female	Male	Female	Male				
84	Spain	2007-2009	Harmonizing Equation of Risk in Mediterraneon countries EXtremadura (HERMEX)	Subnational	25-79	25-79	1,498	1,298	1,491	1,296	Lab	Lab	High-income western	
85	Spain	2008-2010	Study on Nutrition and Cardiovascular Risk in Spain	National	18+	18+	6,803	6,124	6,685	6,029	Lab	Lab	High-income western	
86	Spain	2016-2017	Estudio de Nutricion y Riesgo Cardiovascular en Espana (ENRICA) - Seniors cohort	Subnational	65+	65+	1,739	1,532	542	577	Lab	Lab	High-income western	
87	Suriname	2013-2015	The Healthy Life in Suriname Study (HELISUR)	Subnational	18-70	18-70	722	424	673	406	Lab	Lab	Latin America and the Caribbean	
88	Taiwan	2000	Social Environment and Biomarkers of Aging Study	National	54-80	54-80	433	590	432	589	Lab	Lab	East and southast Asia and the Pacific	
89	Taiwan	2006	Social Environment and Biomarkers of Aging Study	National	53-60	53-60	274	253	202	195	Lab	Lab	East and southast Asia and the Pacific	
90	Thailand	2019-2020	Thailand National Health Examination Survey VI	National	18+	18+	12,375	8,906	12,358	8,891	Lab	Lab	East and southast Asia and the Pacific	
91	Turkey	2012-2013	Turkish Adult Risk Factor Study	National	37+	37+	1,115	1,030	219	206	Lab	Lab	Central Asia, Middle East and north Africa	
92	United Kingdom	2003	Health Survey for England	National	18+	18+	7,823	6,408	447	380	Lab	Lab	High-income western	
93	United Kingdom	2003-2005	Hertfordshire Ageing Study	Subnational	72-82	72-82	151	208	116	169	Lab	Lab	High-income western	
94	United Kingdom	2004-2005	English Longitudinal Study of Ageing Wave 2	National	52-80	52-80	3,716	3,153	1,858	1,569	Lab	Lab	High-income western	
95	United Kingdom	2006-2007	Newcastle 85+ Study	Community	84-86	84-86	526	321	431	283	Lab	Lab	High-income western	5
96	United Kingdom	2008-2009	English Longitudinal Study of Ageing Wave 4	National	50+	50+	1,815	1,590	850	757	Lab	Lab	High-income western	
97	United Kingdom	2006-2010	MRC National Survey of Health and Development	National	60-65	60-65	1,066	966	920	837	Lab	Lab	High-income western	
98	United Kingdom	2008-2012	National Diet and Nutrition Survey	National	18+	18+	1,216	874	903	669	Lab	Lab	High-income western	
99	United Kingdom	2012-2013	English Longitudinal Study of Ageing Wave 6	National	50-88	50-89	1,377	1,214	280	222	Lab	Lab	High-income western	
100	United Kingdom	2013-2014	National Diet and Nutrition Survey	National	18+	18+	514	328	401	276	Lab	Lab	High-income western	
101	United Kingdom	2015-2016	National Diet and Nutrition Survey	National	18+	18+	468	339	364	267	Lab	Lab	High-income western	
102	United Kingdom	2016-2019	National Diet and Nutrition Survey	National	18+	18+	420	301	312	234	Lab	Lab	High-income western	
103	United Kingdom	2018-2019	English Longitudinal Study of Ageing Wave 9	National	50-88	50-85	812	748	294	186	Lab	Lab	High-income western	
104	United States of America	1999-2000	US NHANES 1999-2000	National	18+	18+	2,586	2,547	975	983	Lab	Lab	High-income western	
105	United States of America	2001-2002	US NHANES 2001-2002	National	18+	18+	2,800	2,844	1,126	1,188	Lab	Lab	High-income western	
106	United States of America	2003-2004	US NHANES 2003-2004	National	18+	18+	2,654	2,695	1,047	1,073	Lab	Lab	High-income western	
107	United States of America	2005-2006	US NHANES 2005-2006	National	18+	18+	2,521	2,671	976	1,093	Lab	Lab	High-income western	
108	United States of America	2007-2008	US NHANES 2007-2008	National	18+	18+	3,101	3,064	1,238	1,231	Lab	Lab	High-income western	
109	United States of America	2009-2010	US NHANES 2009-2010	National	18+	18+	3,281	3,174	1,432	1,297	Lab	Lab	High-income western	
110	United States of America	2011-2013	Atherosclerosis Risk in Communities Study (ARIC)	Subnational	67-90	67-90	943	772	880	736	Lab	Lab	High-income western	
111	United States of America	2011-2012	US NHANES 2011-2012	National	18+	18+	2,908	2,894	1,196	1,217	Lab	Lab	High-income western	
112	United States of America	2013-2014	US NHANES 2013-2014	National	18+	18+	3,129	2,916	1,305	1,201	Lab	Lab	High-income western	
113	United States of America	2015-2016	US NHANES 2015-2016	National	18+	18+	3,033	2,885	1,201	1,153	Lab	Lab	High-income western	
114	United States of America	2017-2018	US NHANES 2017-2018	National	18+	18+	2,958	2,839	1,236	1,143	Lab	Lab	High-income western	
115	United States of America	2019-2020	US NHANES 2019-2020	Subnational	18+	18+	1,926	1,878	784	762	Lab	Lab	High-income western	
116	Venezuela	2014-2017	Maracaibo aging study Santa Rosa cohort	Community	37+	37+	291	116	244	97	Lab	Lab	Latin America and the Caribbean	
117	Viet Nam	2008-2009	The Survey on Diabetes and Its Risk Factors	Subnational	25+	25+	1,455	833	1,298	751	Lab	Lab	East and southast Asia and the Pacific	

1 This research uses data from China Health and Nutrition Survey (CHNS). We thank the National Institute of Nutrition and Food Safety, China Center for Disease Control and Prevention, Carolina Population Center (5 R24 HD050924), the University of North Carolina at Chapel Hill, the NIH (R01-HD30880, DK056350, R24-HD050924, and R01-HD38700) and the Fogarty International Center, NIH for financial support for the CHNS data collection and analysis files from 1989 to 2011 and future surveys, and the China-Japan Friendship Hospital, Ministry of Health for support for CHNS 2009.

2 The authors thank the Heinz Nixdorf Foundation [Chairman: Martin Nixdorf; Past Chairman: Dr jur. Gerhard Schmidt], for their generous support of this study. Parts of the study were also supported by the German Research Council (DFG) [DFG project: EI 969/2-3, ER 155/6-1;6-2, HO 3314/2-1;2-2;2-3;4-3, INST 58219/32-1, JO 170/8-1, KN 885/3-1, PE 2309/2-1, SI 236/8-1;9-1;10-1,], the German Ministry of Education and Science [BMBF project: 01EG0401, 01GI0856, 01GI0860, 01GS0820_WB2-C, 01ER1001D, 01GI0205], the Ministry of Innovation, Science, Research and Technology, North Rhine-Westphalia (MIWFT-NRW), the Else Kröner-Fresenius-Stiftung [project: 2015_A119] and the German Social Accident Insurance [DGUV project: FF-FP295]. Furthermore the study was supported by the Competence Network for HIV/AIDS, the deanship of the University Hospital and IFORES of the University Duisburg-Essen, the European Union, the German Competence Network Heart Failure, Kulturstiftung Essen, the Protein Research Unit within Europe (PURE), the Dr. Werner-Jackstädt Stiftung and the following companies: Celgene GmbH München, Imatron/GE-Imatron, Janssen, Merck KG, Philips, ResMed Foundation, Roche Diagnostics, Sarstedt AG&Co, Siemens HealthCare Diagnostics, Volkswagen Foundation. The authors express their gratitude to all study participants of the Heinz Nixdorf Recall (HNR) Study, the personnel of the HNR study center and the EBT-scanner facilities, the investigative group and all former employees of the HNR study. The authors also thank the Advisory Board of the HNR Study: T. Meinertz, Hamburg, Germany (Chair); C. Bode, Freiburg, Germany; P.J. de Feyter, Rotterdam, Netherlands; B. Güntert, Hall i.T., Austria; F. Gutzwiller, Bern, Switzerland; H. Heinen, Bonn, Germany; O. Hess (†), Bern, Switzerland; B. Klein (†), Essen, Germany; H. Löwel, Neuherberg, Germany; M. Reiser, Munich, Germany; G. Schmidt (†), Essen, Germany; M. Schwaiger, Munich, Germany; C. Steinmüller, Bonn, Germany; T. Theorell, Stockholm, Sweden; and S.N Willich, Berlin, Germany.

3 The CARRS Study was funded in part by the National Heart, Lung, and Blood Institute (NHLBI), National Institutes of Health (NIH), Department of Health and Human Services, under Contract No. HHSN268200900026C, the United Health Group, Minneapolis, MN, USA, and by the by the National Heart, Lung, And Blood Institute of the National Institutes of Health under Award Number P01HL154996.

4 The study was supported by the grant of the Ministry of Healthcare of the Republic of Kazakhstan "National Programme for the Introduction of Personalized and Preventive Medicine in The Republic of Kazakhstan (2021–2023)" (Grant number OR12165486).

5 The Newcastle 85+ Study has been funded by the Medical Research Council, Biotechnology and Biological Sciences Research Council, the Dunhill Medical Trust and the National Institute for Health Research School for Primary Care. Parts of the work have also been funded by the British Heart Foundation, Unilever Corporate Research, Newcastle University, NHS North of Tyne (Newcastle Primary Care Trust). Mortality data was obtained from NHS Digital. We acknowledge the operational support of the North of England Commissioning Support Unit, the National Institute for Health Research Clinical Research Network North East and North Cumbria, local general practitioners and their staff. We thank the research nurses, laboratory technicians, data management and clerical team for their work throughout, as well as many colleagues for their expert advice. Thanks are due especially to the study participants and, where appropriate, their families and carers
