# Supplementary material for: Global variation in diabetes diagnosis and prevalence based on fasting glucose and hemoglobin A1c
Source: Nat Med. 2023 Nov 9;29(11):2885–901. doi: 10.1038/s41591-023-02610-2 (PMC10667106; doi:10.1038/s41591-023-02610-2)
Supplement: Supplementary file 2 — Reporting Summary [file 41591_2023_2610_MOESM2_ESM.pdf]

nature portfolio

Corresponding author(s): Majid Ezzati

Last updated by author(s): Sep 20, 2023

Reporting Summary

Nature Portfolio wishes to improve the reproducibility of the work that we publish. This form provides structure for consistency and transparency in reporting. For further information on Nature Portfolio policies, see our Editorial Policies and the Editorial Policy Checklist.

Statistics

For all statistical analyses, confirm that the following items are present in the figure legend, table legend, main text, or Methods section.

n/a	Confirmed	
☒	☐	The exact sample size (*n*) for each experimental group/condition, given as a discrete number and unit of measurement
☒	☐	A statement on whether measurements were taken from distinct samples or whether the same sample was measured repeatedly
☒	☐	The statistical test(s) used AND whether they are one- or two-sided<br>*Only common tests should be described solely by name; describe more complex techniques in the Methods section.*
☐	☒	A description of all covariates tested
☐	☒	A description of any assumptions or corrections, such as tests of normality and adjustment for multiple comparisons
☐	☒	A full description of the statistical parameters including central tendency (e.g. means) or other basic estimates (e.g. regression coefficient) AND variation (e.g. standard deviation) or associated estimates of uncertainty (e.g. confidence intervals)
☒	☐	For null hypothesis testing, the test statistic (e.g. *F*, *t*, *r*) with confidence intervals, effect sizes, degrees of freedom and *P* value noted<br>*Give P values as exact values whenever suitable.*
☐	☒	For Bayesian analysis, information on the choice of priors and Markov chain Monte Carlo settings
☒	☐	For hierarchical and complex designs, identification of the appropriate level for tests and full reporting of outcomes
☒	☐	Estimates of effect sizes (e.g. Cohen's *d*, Pearson's *r*), indicating how they were calculated

Our web collection on statistics for biologists contains articles on many of the points above.

Software and code

Policy information about availability of computer code

Data collection	Processing of secondary data was conducted using the statistical software R (version 4.2.1).
Data analysis	Analyses were conducting using the statistical software R (version 4.2.1) and MultiBUGS (version 2.0). Code for log-binomial model is provided at www.ncdrisc.org and https://doi.org/10.5281/zenodo.8169146.

For manuscripts utilizing custom algorithms or software that are central to the research but not yet described in published literature, software must be made available to editors and reviewers. We strongly encourage code deposition in a community repository (e.g. GitHub). See the Nature Portfolio guidelines for submitting code & software for further information.

Data

Policy information about availability of data

All manuscripts must include a data availability statement. This statement should provide the following information, where applicable:

- Accession codes, unique identifiers, or web links for publicly available datasets
- A description of any restrictions on data availability
- For clinical datasets or third party data, please ensure that the statement adheres to our policy

This is data-pooling study that brings together 117 data sources. Data used in this research are governed by data sharing protocols of participating studies. Contact information for data providers can be obtained from www.ncdrisc.org and https://doi.org/10.5281/zenodo.8169146.

Human research participants

Policy information about studies involving human research participants and Sex and Gender in Research.

Reporting on sex and gender	*Use the terms sex (biological attribute) and gender (shaped by social and cultural circumstances) carefully in order to avoid confusing both terms. Indicate if findings apply to only one sex or gender; describe whether sex and gender were considered in study design whether sex and/or gender was determined based on self-reporting or assigned and methods used. Provide in the source data disaggregated sex and gender data where this information has been collected, and consent has been obtained for sharing of individual-level data; provide overall numbers in this Reporting Summary. Please state if this information has not been collected. Report sex- and gender-based analyses where performed, justify reasons for lack of sex- and gender-based analysis.*
Population characteristics	*Describe the covariate-relevant population characteristics of the human research participants (e.g. age, genotypic information, past and current diagnosis and treatment categories). If you filled out the behavioural & social sciences study design questions and have nothing to add here, write "See above."*
Recruitment	*Describe how participants were recruited. Outline any potential self-selection bias or other biases that may be present and how these are likely to impact results.*
Ethics oversight	*Identify the organization(s) that approved the study protocol.*

Note that full information on the approval of the study protocol must also be provided in the manuscript.

Field-specific reporting

Please select the one below that is the best fit for your research. If you are not sure, read the appropriate sections before making your selection.

☐ Life sciences ☒ Behavioural & social sciences ☐ Ecological, evolutionary & environmental sciences

For a reference copy of the document with all sections, see nature.com/documents/nr-reporting-summary-flat.pdf

Behavioural & social sciences study design

All studies must disclose on these points even when the disclosure is negative.

Study description	We pooled and analysed data from population-based studies that had measured FPG and HbA1c (quantitative data) and collected information on prior diagnosis of diabetes (qualitative data) for adults aged 18 years and over. We reported the proportions of participants who had diagnosed diabetes, and for those without diagnosed diabetes, whether they had elevated FPG (FPG ≥7.0 mmol/L), elevated HbA1c (HbA1c ≥6.5%) or both. We examined the individual-level and study-level factors associated with whether participants with screen-detected diabetes were identified by elevated FPG, elevated HbA1c or elevated levels of both. We tested prediction equations for estimating the probability that a person without diagnosed diabetes at a specific level of FPG had an HbA1c over the clinical threshold for diabetes (HbA1c ≥6.5%), and vice versa.
Research sample	We used all studies collated by the NCD Risk Factor Collaboration that had collected information on whether participants had been previously diagnosed with diabetes, and measured both FPG and HbA1c. In total, we used 117 population-based studies that had data on 601,000 participants aged 18 years or over in 45 countries, of whom 365,000 also had measurements of both FPG and HbA1c.
Sampling strategy	We included studies that had collected data using a probabilistic sampling method with a defined sampling frame. Hence, we included studies with simple random and complex survey designs, and excluded convenience samples and studies whose participants were selected based on factors that might be associated with their diabetes status.
Data collection	We used participant-level data for 601,000 participants from 117 studies. This is an observational study and there was no experiment.
Timing	We used data from surveys with mid-point of data collection period from 2000 to 2021.
Data exclusions	Studies were excluded if they (1) enrolled participants based on health status or cardiovascular risk; (2) were conducted only among ethnic minorities or specific educational, occupational, or other socioeconomic subgroups; (3) recruited participants through health facilities, except studies based on primary care system in high-income and central European countries with universal insurance; (4) had not measured either FPG or HbA1c; (5) had not instructed participants to fast at least for 6 hours prior to FPG measurement; (6) had only measured FPG or HbA1c in the subset of participants who had known diabetes; (7) had measured HbA1c only in a subset of participants selected based on their levels of FPG, and vice versa; (8) had not collected information on prior diagnosis of diabetes; and (9) their mid-year was prior to 2000, before HbA1c assays were widely standardised.<br><br>Participants were excluded if they (1) were pregnant at the time of measurement; (2) had missing sex or age; (3) had missing

	information on prior diagnosis of diabetes; (4) were 18 years of age or younger; (5) had not been measured for FPG or HbA1c by design or data were missing; (6) were from one specific area in one study in Pakistan with high prevalence of thalassemia; (7) were from follow-up rounds of studies that had multiple measurements of the same cohort over time; (8) had FPG <2 or >30 mmol/L or HbA1c <3% or >18%; (9) had implausible combinations of FPG and HbA1c as determined by the method of local outlier factor.
Non-participation	We used all studies that met our inclusion criteria, which were designed to ensure participants of the surveys included were representative of the general population from which each sample was drawn. Information on response rate from individual participating studies is not available to us.
Randomization	Our study is observational, and we did not carry out experiments.

Reporting for specific materials, systems and methods

We require information from authors about some types of materials, experimental systems and methods used in many studies. Here, indicate whether each material, system or method listed is relevant to your study. If you are not sure if a list item applies to your research, read the appropriate section before selecting a response.

Materials & experimental systems

n/a	Involved in the study	
☒	☐	Antibodies
☒	☐	Eukaryotic cell lines
☒	☐	Palaeontology and archaeology
☒	☐	Animals and other organisms
☒	☐	Clinical data
☒	☐	Dual use research of concern

Methods

n/a	Involved in the study	
☒	☐	ChIP-seq
☒	☐	Flow cytometry
☒	☐	MRI-based neuroimaging
